# The OncoSim-Breast Cancer Microsimulation Model

**DOI:** 10.3390/curroncol29030136

**Published:** 2022-03-03

**Authors:** Jean H. E. Yong, Claude Nadeau, William M. Flanagan, Andrew J. Coldman, Keiko Asakawa, Rochelle Garner, Natalie Fitzgerald, Martin J. Yaffe, Anthony B. Miller

**Affiliations:** 1 Canadian Partnership Against Cancer, Toronto, ON M5H 1J8, Canada; natalie.fitzgerald@partnershipagainstcancer.ca; 2 Statistics Canada, Ottawa, ON K1A 0T6, Canada; claude.nadeau@statcan.gc.ca (C.N.); mythinlife@gmail.com (W.M.F.); keikoasakawa0@gmail.com (K.A.); rochelle.garner@statcan.gc.ca (R.G.); 3 British Columbia Cancer Research Institute, Vancouver, BC V5Z 1L3, Canada; andrewjcoldman@gmail.com; 4 Sunnybrook Research Institute, Toronto, ON M4N 3M5, Canada; martin.yaffe@sri.utoronto.ca; 5 Dalla Lana School of Public Health, University of Toronto, Toronto, ON M5T 3M7, Canada; ab.miller@sympatico.ca

**Keywords:** breast cancer, screening, simulation model, costs, effectiveness, incidence, disease progression, natural history

## Abstract

Background: OncoSim-Breast is a Canadian breast cancer simulation model to evaluate breast cancer interventions. This paper aims to describe the OncoSim-Breast model and how well it reproduces observed breast cancer trends. Methods: The OncoSim-Breast model simulates the onset, growth, and spread of invasive and ductal carcinoma in situ tumours. It combines Canadian cancer incidence, mortality, screening program, and cost data to project population-level outcomes. Users can change the model input to answer specific questions. Here, we compared its projections with observed data. First, we compared the model’s projected breast cancer trends with the observed data in the Canadian Cancer Registry and from Vital Statistics. Next, we replicated a screening trial to compare the model’s projections with the trial’s observed screening effects. Results: OncoSim-Breast’s projected incidence, mortality, and stage distribution of breast cancer were close to the observed data in the Canadian Cancer Registry and from Vital Statistics. OncoSim-Breast also reproduced the breast cancer screening effects observed in the UK Age trial. Conclusions: OncoSim-Breast’s ability to reproduce the observed population-level breast cancer trends and the screening effects in a randomized trial increases the confidence of using its results to inform policy decisions related to early detection of breast cancer.

## 1. Introduction

Rapidly emerging knowledge in breast cancer control has put pressure on the health system for the adoption of new technologies and policies. Randomized trials are the gold standard of evidence to introduce new interventions in clinical practice and public health; however, such evidence is not always relevant for informing policy decisions because the context of the interventions evolves quickly, compared with the time that elapses between the design of a trial and the availability of its results. For example, most breast cancer screening randomized trials were from the era before breast cancer adjuvant treatment was available and used film-screen mammography [1,2]; breast cancer survival has since vastly improved [3], and digital mammography has superseded film-screen mammography. A cancer simulation model can help integrate evidence from multiple sources and make them more relevant to inform contemporary clinical and policy decisions. 

Several groups have developed sophisticated cancer-specific models based on the natural history of cancer that can be revised for additional analyses and incorporate knowledge from experts in different areas [4]. An example includes breast cancer models developed by the CISNET breast cancer working group, where the models have been used extensively to investigate emerging issues in breast cancer control and to inform debates on topics such as breast density legislation in the US [4]. OncoSim-Breast is an example of such a model but developed for the Canadian population using Canadian data, whenever applicable. It is the only breast cancer model of this nature, i.e., a microsimulation model developed for informing various breast cancer control questions, in Canada. When compared with the CISNET breast cancer models, OncoSim-Breast is unique in that it is available at no charge to users in the public sector. Breast cancer is the latest addition to OncoSim’s suite of cancer models. The validation and applications of OncoSim colorectal, cervical, and lung cancers have been described previously [5,6,7,8,9,10,11,12,13,14,15,16,17,18,19]. Briefly, these models were developed using country-specific data, whenever available, and were calibrated to match the key output in the national cancer registry. These models were also used to inform the development and revisions of clinical guidelines, and the design and implementation of cancer screening programs [13]. The primary objective of OncoSim-Breast is to investigate emerging issues related to breast cancer control in Canada. This work builds on a strong foundation of analyses performed over a decade ago to estimate the impact of diagnostic and therapeutic approaches to non-metastatic breast cancer in Canada, using the Statistics Canada POHEM mathematical model [20]. The present paper has two goals. First, it aims to describe the key assumptions in OncoSim-Breast. Secondly, it compares OncoSim-Breast’s projections with observed data in the Canadian Cancer Registry, projected breast cancer mortality estimates in the Canadian Vital Statistics, and the observed screening effects in a randomized trial.

## 2. Materials and Methods

### 2.1. OncoSim-Breast

**Figure 1:**
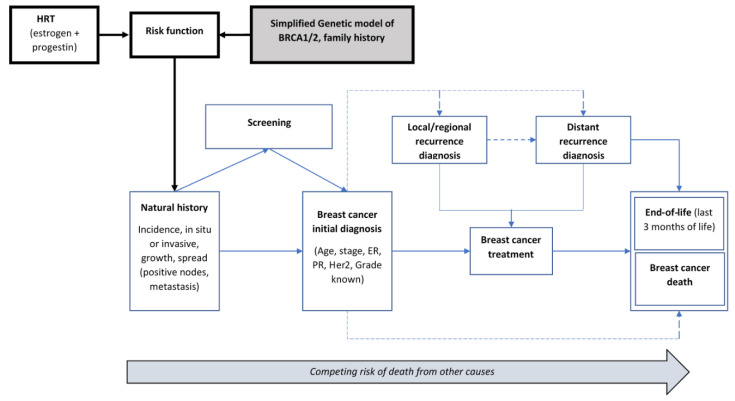
Schematic diagram of the OncoSim-Breast model.

**Figure 2:**
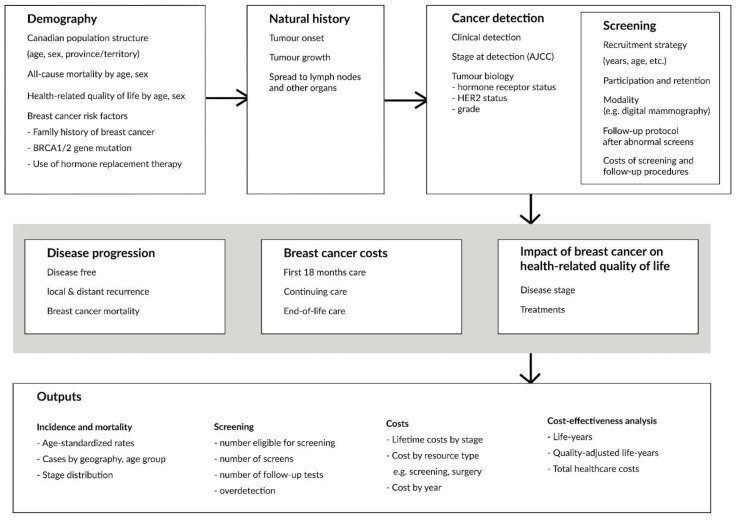
Model inputs and outputs.

The OncoSim-Breast mathematical simulation model combines inputs (demography, the natural history of tumour development and progression, screening, cancer costs, and quality of life) to project population-level outcomes, such as breast cancer incidence and mortality, screening outcomes, stage and age at diagnosis, life years, quality-adjusted life years, lifetime breast cancer costs, and screening or follow-up procedure costs (Figure 1 and Figure 2, Table 1).

### 2.2. Demography

OncoSim simulates one individual at a time, replicating the age and sex distributions and all-cause mortality of the population in each province and territory in Canada (Supplemental File S1: Section 1). Each simulated individual has attributes, such as demography (sex, province/territory), and breast cancer-related risk factors (BRCA1/2 gene mutation, family history, and exposure to hormone replacement therapy; Supplemental File S1: Table S1). 

### 2.3. Natural History

OncoSim-Breast simulates the onset, growth, and spread of tumours, both invasive cancer and Ductal Carcinoma in situ (DCIS) (Supplemental File S1: Section 2). In the model, invasive tumours can develop without an apparent prior in situ component, because they became invasive before reaching the 2 mm threshold of our simulation. In situ and invasive tumours are allowed to develop and grow independently of each other. Thus, a woman could have one of the four outcomes: (1) in situ disease, (2) an invasive tumour, (3) in situ disease that becomes invasive and evolves independently of the initial in situ component, or (4) no breast tumour at all. The development, tumour biology, growth, and clinical detection of breast cancers, both invasive cancer and DCIS, were calibrated from inputs in the University of Wisconsin Breast Cancer Epidemiology Simulation Model (“Wisconsin Breast model”) [32] to match the incidence of cancer by age group and year in the National Cancer Incidence Reporting System (1969–1991), Canadian Cancer Registry (1992–2013) and Canadian Breast Cancer Screening Database (2007–2008). 

Tumour onset: In OncoSim-Breast, tumours start from 2 mm, based on the probable minimum size detectable by mammography screening and similar to the Wisconsin Breast model. The probability of tumour onset varies by age and years (Supplemental File S1: Figure S1). In addition, the risk increases if a woman has any of the breast cancer-related risk factors (BRCA1/2 mutation, family history of breast cancer, or exposure to hormone replacement therapy; Supplemental File S1: Tables S3 and S4); if a woman has previously had a DCIS tumour, she is also more likely to have invasive cancer (see equation in Supplemented Methods).

Tumour growth: In the model, tumours grow according to the time since tumour onset, the presence of BRCA1/2 gene mutation, tumour type (DCIS or invasive), and tumour aggressiveness (Supplemental File S1: Figure S2). All tumours were assumed to grow according to a Gompertz distribution that gives the tumour diameter (d) in cm as a function of years since tumour onset (t), scaled according to the maximum diameter allowed for a tumour type, according to the following equation:

$$d\left(t\right)=d_{0}{\left(\frac{d_{\max}}{d_{0}}\right)}^{\left(1-e^{-\alpha t}\right)}\;$$

where 

$d_{0}$ is the diameter of the tumour at occult onset (0.2 cm);

$d_{\max}$ is the maximum size the tumour is allowed to reach;

α represents the tumour growth rate estimated through model fitting;

t represents the years since tumour onset.

The breast tumour growth equation coefficients were calibrated from the Wisconsin Breast model’s parameters to match stage-specific incidence data in Canadian Cancer Registry (1992–2013) and Canadian Breast Cancer Screening Database (2007–2008) and various other targets (Supplemental File S1: Table S5). Supplemental File S1: Figure S2 shows the growth curves by tumour type and class for a mean growth rate and mean max size. 

Tumour spread: An invasive tumour can spread into lymph nodes and beyond the breast. The spread to other lymph nodes is determined by the size and growth rate of the tumour and time since tumour onset as follows:

$$\lambda \left(t\right)={\;\mu }_{N}\left\{b_{1}+b_{2}V\left(t\right)+b_{3}V^{\prime }\left(t\right)\right\}$$

where

$\mu_{N}$ is the propensity to generate positive nodes. It is drawn from a gamma distribution (mean and variance in Supplemental File S1: Table S5) at the time of tumour onset to allow for heterogeneity; 

b_1_, b_2_, b_3_ are coefficients estimated through calibration of natural history (Supplemental File S1: Table S6);

V(t) denotes the volume of the spherical tumour;

V′(t) denotes the growth rate of the volume, and is the derivative of V(t);

t represents the age of the tumour, i.e., years since oncogenesis.

The equation was adopted from the CISNET–Wisconsin model and was calibrated to match positive node data in Canadian Cancer Registry (1992–2013) and Canadian Breast Cancer Screening Database (2007–2008). When calibrating the model, we assumed that subsequent non-invasive tumours cannot develop into an invasive tumour once a woman has developed an invasive tumour. However, there is no limit in the number of positive nodes an invasive tumour could generate. 

The tumour size and the number of lymph nodes affected then determine if invasive cancer spreads beyond the breast (metastasis). The following equation governs the metastasis rate of an invasive tumour:

$$\mathrm{Hazard}\;\mathrm{of}\;\mathrm{metastasis}=\mu_{M}\times k\left(\mathrm{tumour}\;\mathrm{size},\;\mathrm{number}\;\mathrm{of}\;\mathrm{positive}\;\mathrm{nodes}\right)$$

where

µ_M_ is the propensity for metastasis. It is drawn from a gamma distribution (mean and variance in Supplemental File S1: Table S5) at the time of tumour onset to allow for heterogeneity; 

k is an annual metastasis hazard estimated through model calibration. It is a function of tumour size and the number of positive nodes (Supplemental File S1: Table S7). 

The hazard was calibrated to match stage-specific incidence data in Canadian Cancer Registry (1992–2013) and the Canadian Breast Cancer Screening Database (2007–2008). The overall rate of metastasis is the cumulative metastasis rate of all the invasive tumours in a person. For example, if a woman has three invasive tumours, her annual metastasis hazard is the sum of the metastasis hazard of the three tumours.

### 2.4. Cancer Detection, Staging, and Tumour Biology

Cancer detection: The probability of cancer being detected depends on the tumour size and the number of tumours. If a woman has multiple tumours, we assumed her cancer detection probability is the sum of the clinical detection probability of the individual tumours (Supplemental File S1: Table S8). Clinical detection probability for a tumour was calibrated from the inputs in the Wisconsin Breast model to match the incidence data in the National Cancer Incidence Reporting System (1969–1991) and the Canadian Cancer Registry (1992–2013). The hazards were interpolated linearly for in-between sizes. 

Staging: The stage at detection uses the American Joint Committee on Cancer (AJCC) classification of tumour size (T), nodal status (N), and metastasis (M) (Supplemental File S1: Table S9). The tumour size and nodal status at detection are estimated using the tumour size and number of positive nodes generated from the natural history component and age. First, the model determines if a tumour is a T4 tumour; the probability of a T4 tumour (have extended to the chest and/or skin) is a function of tumour size T* and the number of positive nodes N* (Supplemental File S1: Table S10). Next, it estimates T based on T* for non-T4 tumours. 

N: The model assigns nodal status (N in TNM) at the time of detection from a distribution that depends on the number of positive nodes N* and T, fitted using the Canadian Cancer Registry data (Supplemental File S1: Table S11). 

Tumour biology: To simplify the model, OncoSim assigns tumour biology (hormone receptor status, HER2neu status, and grade) once tumour has been detected. The joint distribution of these biological factors was estimated from the Canadian Cancer Registry by tumour size (Tis, T1a, T1b, T1c, T2, T3, T4), nodal involvement (N0, N1, N1mi, N2, N3), metastatic status and age of women at tumour detection (10-year age groups) (Supplemental File S2). Women with BRCA1/2 gene mutation have different distributions of tumour biology than women without BRCA1/2 gene mutation [33]. Women who used hormone replacement therapy have different tumour grades [34]. 

### 2.5. Disease Progression

Upon cancer detection, the model draws time to disease progression (recurrence and breast cancer death), based on stage, tumour biology, age at diagnosis, and detection method (clinically or screening). A woman will die from breast cancer if the simulated time to breast cancer death is sooner than the simulated time to non-breast cancer death. We modelled disease progression using data from a cohort of women diagnosed with breast cancer in British Columbia between 2006 and 2009 and followed up until 2014. We fitted the stage-specific outcomes data (diagnosis to local recurrence, diagnosis to distant recurrence, local recurrence to distant recurrence, etc.) to Weibull regression models, controlling for the number of years from diagnosis, age, grade, hormone status, her2-neu status, screening status, and the variables’ interactions (Supplemental File S1: Tables S12–S16). Stage-specific recurrence risks and breast cancer survival outcomes were estimated using data from the British Columbia Cancer Agency because comprehensive staging data only became available recently in the Canadian Cancer Registry. To capture provincial differences in stage-specific survival, the model applies province-specific relative risks, estimated from more recent data in the Canadian Cancer Registry to the British Columbia survival curves (Supplemental File S1: Table S17). 

### 2.6. Screening

In OncoSim, screening can detect tumours earlier than they would have been detected clinically. The survival from time of screen-detection to breast cancer death includes lead time and net survival benefit (Supplemental File S1: Figure S3). Neither lead time nor net survival benefit is input to the model: rather, these can be estimated from the model output. Survival models were calibrated to match the observed survival data from a cohort of women diagnosed with breast cancer in British Columbia in 2006–2009; the survival data from these women were available up to 2014. Screen detection also leads to a stage shift that contributes to the survival benefit. The model reports overdetection (cancers that would not otherwise present clinically) as an output.

For evaluating screening strategies or related performance, the model allows users to create different screening strategies and scenarios by modifying the following input parameters: screening program recruitment strategy (e.g., start/end age and years); screening participation and retention; screening frequency; screening modality (e.g., digital mammography); sensitivity and specificity of screening; follow-up protocol after abnormal screening results; and costs of screening and follow-up procedures.

The model also includes historical breast screening trends in Canada (starting in 1986) to match the observed screening patterns reported in the screening programs in 2007–2012. Screening interventions can vary by family history and BRCA1/2 gene mutation. The model includes different screening modalities and allows their performance to vary by tumour size, age group, and screen sequence (Supplemental File S1: Figure S5 and Table S18). Women with an abnormal mammogram receive additional workups, such as diagnostic imaging, biopsy, and fine-needle aspiration. The model includes costs of screening and follow-up procedures from the perspective of a public healthcare payer, such as the Ministry of Health (Supplemental File S1: Table S13). 

### 2.7. Breast Cancer Costs

The model included healthcare costs associated with breast cancer from the perspective of a public healthcare payer, such as the Ministry of Health (Supplemental File S1: Section 6). The costs included breast cancer surgery, radiation treatment, chemotherapy, imaging tests and oncology physician fees, acute hospitalizations, emergency department visits, home care, long-term care, complex continuing care, and others. The model captures lifetime costs of breast cancer across three phases of care (first 18 months after diagnosis, continuing care, and terminal care), a similar approach as that used in other established breast cancer simulation models [3].

### 2.8. Health-Related Quality of Life

To calculate quality-adjusted life years after an individual is diagnosed with breast cancer, the model multiplies the duration of each health state with age- and sex-specific preference scores for the Canadian population and breast cancer-specific health state utilities (upon cancer diagnosis) (Supplemental File S1: Tables S21 and S22) [30,31]. When an individual is in several health states at the same time, we assumed the utility score is multiplicative [35].

### 2.9. Model Validation

We validated our model in three ways using OncoSim version 3.3.6. First, for face validation, we plotted the projected incidence and stage distribution of breast cancer in Canada and the observed data in the Canadian Cancer Registry (1992–2017). Second, as another face validation exercise, we compared OncoSim’s projected breast cancer deaths in 2018 and the latest breast cancer death data in the Canadian Vital Statistics [36]. Lastly, as an external validation exercise, we simulated the screening strategies of the UK Age trial [37,38] in OncoSim to compare OncoSim’s projected impact of breast cancer screening on incidence and mortality with the observed effects in the trial. 

The UK Age trial is a randomized trial that compared annual screening in women aged 40–49 years with usual care in the UK in the 1990s [37]. To compare our results with other established breast cancer simulation models, we set up our simulation following their methods when they compared their predictions against the UK trial results (details of the simulation have been reported in another paper) [39]. Briefly, we simulated a cohort of women born in 1950–1957 to match the birth cohort in the UK Age trial in two scenarios: (1) no screening and (2) annual screening for women age 40–49. In the screening scenario, we calibrated the rescreening rate to the average number of mammograms per woman in the Age trial (4.8) [39]. For each scenario, we estimated the incidence of breast cancer and breast cancer deaths in women aged 40–49 years. We then compared OncoSim-Breast’s projected incidence of breast cancer (DCIS and invasive cancers) with the trial’s mean estimate and its 95% confidence interval. For breast cancer mortality, we compared the mortality reduction ratio from OncoSim-Breast with the trial’s mean estimate and 95% confidence interval at the 10-year and 17-year follow-up. We chose to compare rate ratios rather than rates because the populations were different: volunteers in the UK Age trial vs. Canadian population. In this simulation, we did not adjust the natural history to match the UK population, and we did not change the all-cause mortality variable for the UK population. 

## 3. Results

**Figure 3:**
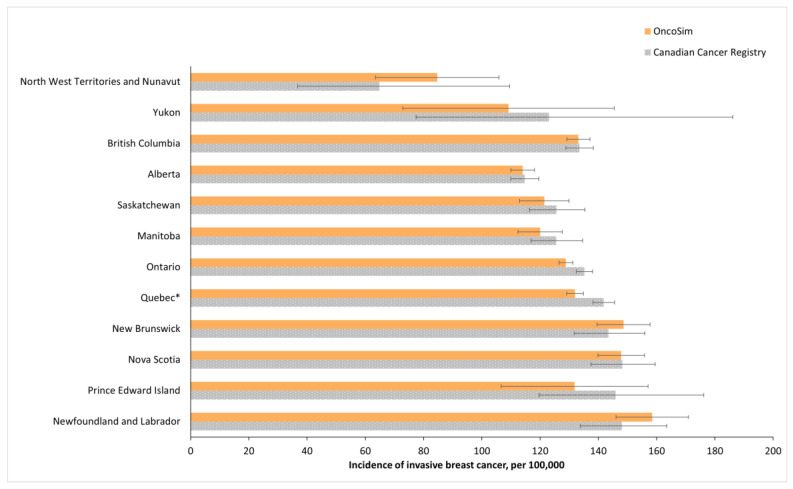
Incidence of invasive breast cancer (per 100,000 women), average per year (2008–2017), by province, OncoSim-Breast vs. Canadian Cancer Registry (CCR). * Data from Quebec were available only in 2008–2010 in the Canadian Cancer Registry because Quebec switched to a different cancer reporting system after 2010. Error bars represent the 95% confidence intervals.

**Figure 4:**
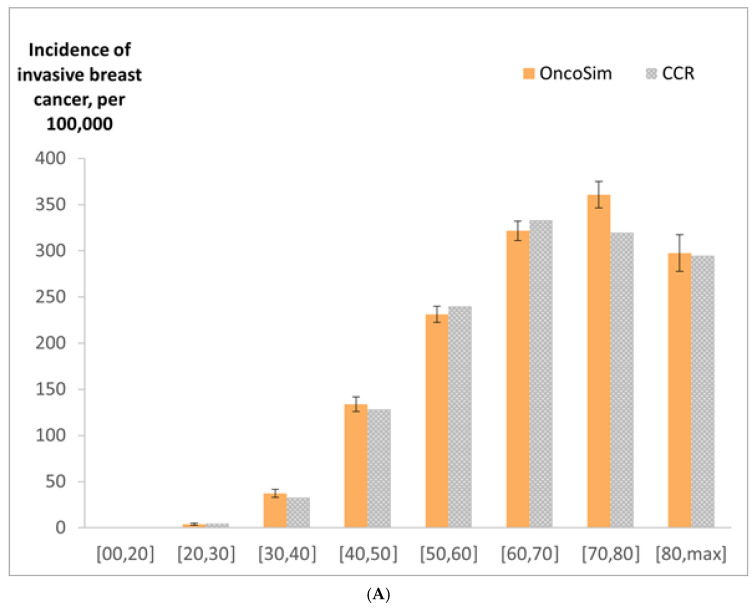

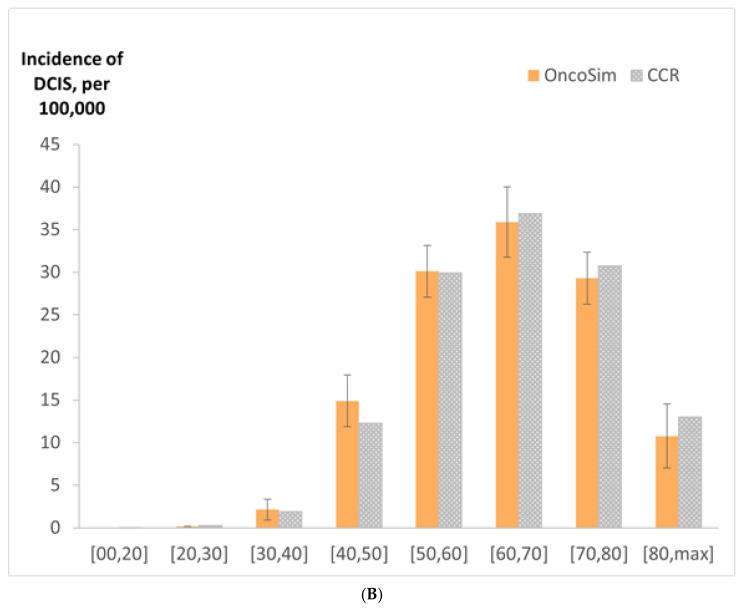
(**A**) Incidence of invasive breast cancer (per 100,000 women) by age group in 1992–2013, OncoSim-Breast vs. Canadian Cancer Registry (CCR); (**B**) incidence of ductal carcinoma in situ (per 100,000 women) by age group in 1992–2013, OncoSim-Breast vs. Canadian Cancer Registry (CCR). Error bars represent the 95% confidence intervals.

**Figure 5:**
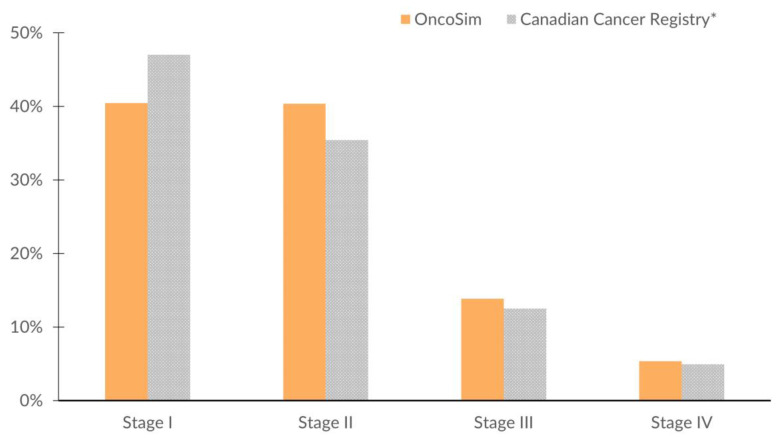
Distribution of breast cancer by stage at diagnosis, females, Canada, 2011–2015, OncoSim-Breast vs. Canadian Cancer Registry. * The Canadian Cancer Registry did not include data from Quebec in 2011–2015 because Quebec switched to a different cancer reporting system after 2010.

OncoSim’s projected breast cancer incidence and deaths at the national level were close to the observed data in recent years (projected incidence in Figure 3). OncoSim’s projected a breast cancer death rate of 27 per 100,000 women in 2018 and the Vital Statistics reported 28 deaths per 100,000 women [36]. When projecting breast cancer incidence by province/territory in recent years (2008–2017), OncoSim’s estimates were also close to the observed data for most jurisdictions (Figure 3). Its projections were within the confidence intervals of the Canadian Cancer Registry data for all provinces and territories, except the two larger provinces (Quebec and Ontario), where its projections were slightly lower. OncoSim’s projected age trend in the incidence of invasive breast cancer and DCIS was also similar to that in the Registry (Figure 4A,B) in 1992–2013. When comparing the projected incidence for specific age groups, OncoSim’s projection was slightly higher in women aged 70–79 years in 1992–2013. For stage distribution, OncoSim’s projected that 80% of breast cancer cases diagnosed in 2011–2015 were earlier stage cases (stage I and II), whereas the observed data in the Canadian Cancer Registry reported 82% (Figure 5). 

In our external validation exercise, we estimated the effects of annual breast cancer screening in women aged 40–49 years. OncoSim’s projections were within the confidence intervals of the observed results from the UK Age trial (Table 2). When estimating the mortality reduction in breast cancer screening, OncoSim estimated a smaller effect than the Age trial at the 10-year follow-up, but the estimates were more similar at the 17-year follow-up. OncoSim’s projections were almost identical to the average mortality reduction predicted by the five CISNET breast cancer models at the 10- and 17-year follow-up [39].

## 4. Discussion

This paper provides an overview of OncoSim-Breast inputs, assumptions, breast cancer cost projections, and model validation results. When projecting incidence, mortality, and stage at diagnosis of breast cancer, OncoSim-Breast’s estimates were close to the estimates reported in the Canadian Cancer Registry and Vital Statistics. In addition, OncoSim-Breast’s ability to reproduce the observed effects of annual breast cancer screening in a randomized screening trial increases the confidence of using the model results to inform breast cancer screening-related policy decisions. When simulating the effects of breast cancer screening in women aged 40–49 years on breast cancer mortality, OncoSim’s projections were almost identical to the average projections from the CISNET breast cancer models [39]. 

Building upon the experience of other OncoSim models and another established breast cancer microsimulation model^3^, OncoSim-Breast was developed using Canadian data. While the model has many potential applications, its primary purpose was to evaluate the impact of interventions related to early detection, such as promoting breast cancer awareness through professional and public education and screening. For screening, the model has many detailed outputs for informing policy decisions, including the harm of screening (e.g., false positives and overdetection), healthcare costs, and benefits (life years gained, cancer incidence and mortality, and quality-adjusted life years). Jurisdictions planning the implementation of population-based breast cancer screening can compare the impact of different screening strategies. For jurisdictions that have an organized breast cancer screening program in place, OncoSim-Breast could help investigate emerging issues such as increasing false positives and customizing screening protocols based on different risk factors. In addition, jurisdictions can use the model to assess the impact of service disruptions during the COVID-19 pandemic [41]. For example, they can estimate the impact of pausing screening for various time intervals on the stage of diagnosis and breast cancer deaths. They can also compare the impact of different strategies for restoring screening programs on downstream resources, such as follow-up diagnostics, biopsies, and surgeries.

## 5. Limitations

This paper has several limitations. First, OncoSim is a simulation model built using the best available data; the accuracy of projections depends on the quality of data input and the validity of assumptions. To address the issue of rapidly emerging evidence, OncoSim-Breast allows users to modify the inputs and assumptions. Second, our comparison of OncoSim-Breast’s projections with more recent Canadian Cancer Registry data was limited by the availability and quality of data in the Registry. Third, our simulation of the UK Age trial was an exploratory external validation exercise; we did not calibrate the model to reflect the use of single-view mammography in AGE or to match the historically poorer breast cancer outcomes at that time. Fourth, OncoSim-Breast was built to be a multi-purpose breast cancer simulation tool and could simulate many scenarios; therefore, it would not be feasible to validate all its possible projections against observed data. To ensure OncoSim-Breast’s relevance for supporting policy decisions, the team compares OncoSim-Breast’s projections with emerging real-world data and refines the model based on new evidence, on an ongoing basis. In the upcoming releases, examples of further enhancements will include adding emerging data on new screening modalities and other factors that might affect screening performance, such as breast density and polygenic risk scores. Fifth, the model does not consider the impact of comorbidity on breast cancer survival. Finally, OncoSim-Breast focuses on breast cancer in women only.

## 6. Conclusions

OncoSim-Breast is a natural history-based simulation model developed using Canadian cancer incidence, mortality, screening program, and cost data. It reproduces breast cancer trends in the Canadian Cancer Registry, breast cancer mortality in the Vital Statistics, and the breast cancer screening effects observed in a randomized screening trial.

## Figures and Tables

**Table 1:** Model inputs data sources.

Model Inputs	Estimates	Data Sources
**Demography**		
Canadian population structure (age, sex, province/territory)		Statistics Canada Demography Division
All-cause mortality by age, sex		Statistics Canada Demography Division
Breast cancer risk factors − Proportion of women with BRCA1/2 gene mutation − Breast cancer family history distribution − Hormone replacement therapy use	Supplemental File S1: Table S1	Anglian Breast Cancer Study group [21] Canadian National Breast Screening Study (CNBSS) [22] National Population Health Survey (1994–2010) [23]
**Natural history**		
Rate of occult tumour onset (oncogenesis)	Supplemental File S1: Figure S1	Calibrated from the input parameters in the University of Wisconsin Breast Cancer Model [24] to match the incidence data in the cancer registry *.
Distribution of tumour type (DCIS vs. invasive) by age	Supplemental File S1: Table S2	
Relative risk of developing occult tumour based on BRCA1/2 gene mutation and breast cancer family history	Supplemental File S1: Table S3	Calibrated from Singletary SE (2003) [25] to match the incidence data in the cancer registry *
Relative risk of developing occult tumour based on hormone therapy use	Supplemental File S1: Table S4	Calibrated to match the results of a study reporting the impact of hormone therapy use on breast cancer risk [26]
Tumour growth	$d\left(t\right)=d_{0}{\left(\frac{d_{\max}}{d_{0}}\right)}^{\left(1-e^{-\alpha t}\right)}$ Supplemental File S1: Table S5	Calibrated from the Wisconsin Breast model’s parameters [24] to match stage-specific incidence data in the Canadian Cancer Registry (1992–2013) and Canadian Cancer Screening Database (2007–2008)
Tumour spread to other lymph nodes, hazard	$\lambda \left(t\right)={\;\mu }_{N}\left\{b_{1}+b_{2}V\left(t\right)+b_{3}V^{\prime }\left(t\right)\right\}$ Supplemental File S1: Tables S5 and S6	
Metastasis hazard	$\begin{array}{ll} \mathrm{Hazard}\;\mathrm{of}\;\mathrm{metastasis}\\ & =\mu_{M}\\ & \times k\left(\mathrm{tumour}\;\mathrm{size},\;\mathrm{number}\;\mathrm{of}\;\mathrm{positive}\;\mathrm{nodes}\right) \end{array}$ Supplemental File S1: Tables S5 and S7	Calibrated to match stage-specific incidence data in Canadian Cancer Registry (1992–2013) and Canadian Breast Cancer Screening Database (2007–2008).
**Cancer detection**		
Probability of clinical detection by tumour size	Supplemental File S1: Table S8	Calibrated from the input parameters in the University of Wisconsin Breast Cancer Model [24] to match the incidence data in the cancer registry *.
Stage distribution at detection	Supplemental File S1: Tables S9–S11	Canadian Cancer Registry *
Breast tumour biology	Joint distribution of hormone receptor status, HER2neu status, and grade at detection, by tumour size, nodal involvement, metastatic status, and age of women at tumour detection (Supplementary File S2)	Canadian Cancer Registry *
**Disease progression**		
Stage-specific recurrence and survival risks	Supplemental File S1: Tables S12–S16	Unpublished data from British Columbia †
Province/territory-specific relative risk of breast cancer survival	Supplemental File S1: Table S17	Canadian Cancer Registry *
**Screening**		
Sensitivity and specificity of mammography	Supplemental File S1: Figure S5 and Table S18	
Cost of follow-up procedures for abnormal screen results	Supplemental File S1: Table S19	Ontario Breast Screening Program 2011, Canadian Breast Cancer Screening Database 2004–2008 and Ontario Health Insurance fee schedules [27,28]
Breast cancer costs	Supplemental File S1: Section 6	Retrospective administrative database analysis using Ontario data, Ontario Health Insurance Program schedule of benefits, and end-of-life costing study of breast cancer patients [27,29]
Age-specific health state utilities–Canadian general population	Supplemental File S1: Table S21	[30]
Breast cancer-specific preference score	Supplemental File S1: Table S22	[31]

* National Cancer Incidence Reporting System (1969–1991) and the Canadian Cancer Registry (1992–2013). † Observed survival data from a cohort of women diagnosed with breast cancer in British Columbia in 2006–2009; the survival data from these women were available up to 2014.

**Table 2:** OncoSim’s projections vs. the observed estimates from the UK Age trial and predictions from the CISNET models.

	OncoSim	Age Trial	CISNET Models *
Detection of invasive breast cancer	16% more	10% (95% CI: 0.95 to 1.21) [40]	N/A
Breast cancer death reduction at 10-year follow-up	15%	25% (95% CI, 3% to 42%) [37]	15% (range, 13% to 17%)
Breast cancer death reduction at 17-year follow-up	15%	12% (95% CI: −4% to 26%) [38,39]	13% (range, 10% to 17%)

* Five breast cancer models in the CISNET consortium reported their projections. Here, we report the average and range of predictions from the five models [39].

## Data Availability

Detailed data about model input parameters and projections are available on the OncoSim web application via https://www.partnershipagainstcancer.ca/tools/oncosim/ (accessed on 22 February 2022).

## Supplementary Materials

The following supporting information can be downloaded at: www.mdpi.com/article/10.3390/curroncol29030136/s1, Supplemental File S1–Supplementary Methods, Figures and Tables [42,43,44,45,46,47]; Supplementary File S2–Tumour biology joint distribution.

## References

[1] Miller A.B., To T., Baines C.J., Wall C. (2002). The Canadian National Breast Screening Study-1: Breast cancer mortality after 11 to 16 years of follow-up: A randomized screening trial of mammography in women age 40 to 49 years. Ann. Intern. Med..

[2] Nyström L., Bjurstam N., Jonsson H., Zackrisson S., Frisell J. (2017). Reduced breast cancer mortality after 20+ years of follow-up in the Swedish randomized controlled mammography trials in Malmö, Stockholm, and Göteborg. J. Med. Screen..

[3] Saadatmand S., Bretveld R., Siesling S., Tilanus-Linthorst M.M.A. (2015). Influence of tumour stage at breast cancer detection on survival in modern times: Population based study in 173,797 patients. BMJ.

[4] Alagoz O., Berry D.A., de Koning H.J., Feuer E.J., Lee S.J., Plevritis S.K., Schechter C.B., Stout N.K., Trentham-Dietz A., Mandelblatt J.S. (2018). Introduction to the Cancer Intervention and Surveillance Modeling Network (CISNET) breast cancer models. Med. Decis. Mak..

[5] Coldman A., Flanagan W., Nadeau C., Wolfson M., Fitzgerald N., Memon S., Gauvreau C., Miller A., Earle C. (2017). Projected effect of fecal immunochemical test threshold for colorectal cancer screening on outcomes and costs for Canada using the OncoSim microsimulation model. J. Cancer Policy.

[6] Coldman A., Pader J., Gauvreau C., Memon S., Fitzgerald N., Flanagan W., Nadeau C., Earle C., Wolfson M., Miller A. (2018). Simulating results from trials of sigmoidoscopy screening using the OncoSim microsimulation model. J. Cancer Policy.

[7] Coldman A., Phillips N., Brisson J., Flanagan W., Wolfson M., Nadeau C., Fitzgerald N., Miller A. (2015). Using the Cancer Risk Management Model to evaluate colorectal cancer screening options for Canada. Curr. Oncol..

[8] Evans W., Gauvreau C., Flanagan W., Memon S., Fitzgerald N., Goffin J., Miller A. (2017). Costs and Cost-Effectiveness of Smoking Cessation within an Organized CT Lung Cancer (LC) Screening Program. J. Thorac. Oncol..

[9] Evans W., Gauvreau C., Memon S., Goffin J., Lacombe J., Wolfson M., Fitzgerald N., Miller A. (2017). Potential Health and Economic Consequences of Organized vs Opportunistic Lung Cancer Screening in Canada. J. Thorac. Oncol..

[10] Evans W.K., Flanagan W.M., Miller A.B., Goffin J.R., Memon S., Fitzgerald N., Wolfson M. (2016). Implementing low-dose computed tomography screening for lung cancer in Canada: Implications of alternative at-risk populations, screening frequency, and duration. Curr. Oncol..

[11] Fitzgerald N.R., Flanagan W.M., Evans W.K., Miller A.B., Canadian Partnership against Cancer Cancer Risk Management Lung Cancer Woring Grooup (2015). Eligibility for low-dose computerized tomography screening among asbestos-exposed individuals. Scand. J. Work Environ. Health.

[12] Flanagan W.M., Evans W.K., Fitzgerald N.R., Goffin J.R., Miller A.B., Wolfson M.C. (2015). Performance of the cancer risk management model lung cancer screening module. Health Rep..

[13] Gauvreau C.L., Fitzgerald N.R., Memon S., Flanagan W.M., Nadeau C., Asakawa K., Garner R., Miller A.B., Evans W.K., Popadiuk C.M. (2017). The OncoSim model: Development and use for better decision-making in Canadian cancer control. Curr. Oncol..

[14] Goffin J.R., Flanagan W.M., Miller A.B., Fitzgerald N.R., Memon S., Wolfson M.C., Evans W.K. (2016). Biennial lung cancer screening in Canada with smoking cessation—Outcomes and cost-effectiveness. Lung Cancer.

[15] Goffin J.R., Flanagan W.M., Miller A.B., Fitzgerald N.R., Memon S., Wolfson M.C., Evans W.K. (2015). Cost-effectiveness of Lung Cancer Screening in Canada. JAMA Oncol..

[16] Lacombe J., Gauvreau C., Memon S., Popadiuk C., Flanagan W., Nadeau C., Coldman A.J., Wolfson M., Miller M. (2016). Exploring the health outcomes of various pan-Canadian cervical cancer screening programs using microsimulation modeling. Am. J. Epidemiol..

[17] Miller A.B., Gribble S., Nadeau C., Asakawa K., Flanagan W.M., Wolfson M., Coldman A., Evans W.K., Fitzgerald N., Lockwood G. (2015). Evaluation of the natural history of cancer of the cervix, implications for prevention. The Cancer Risk Management Model (CRMM)—Human papillomavirus and cervical components. J. Cancer Policy.

[18] Popadiuk C., Coldman A., Memon S., Fitzgerald N., Gribble S., Lockwood G., Wolfson M., Miller A. (2016). Comparing the health and economic impacts of cervical cancer screening strategies using the Cancer Risk Management Model (CRMM). Gynecol. Oncol..

[19] Popadiuk C., Gauvreau C., Bhavsar M., Nadeau C., Asakawa K., Flanagan W., Wolfson M., Coldman A., Memon S., Fitzgerald N. (2016). Using the Cancer Risk Management Model to evaluate the health and economic impacts of cytology compared with human papillomavirus DNA testing for primary cervical cancer screening in Canada. Curr. Oncol..

[20] Hennessy D.A., Flanagan W.M., Tanuseputro P., Bennett C.J., Tuna M., Kopec J.A., Wolfson M.C., Manuel D.G. (2015). The Population Health Model (POHEM): An overview of rationale, methods and applications. Popul. Health Metr..

[21] Anglian Breast Cancer Study Group (2000). Prevalence and penetrance of BRCA1 and BRCA2 mutations in a population-based series of breast cancer cases. Br. J. Cancer.

[22] Miller A.B., Baines C.J., To T., Wall C. (1992). Canadian National Breast Screening Study: 1. Breast cancer detection and death rates among women aged 40 to 49 years. CMAJ.

[23] Statistics Canada National Population Health Survey. https://www150.statcan.gc.ca/n1/en/catalogue/82F0001X.

[24] National Cancer Institute Cancer Intervention and Surveillance Modeling Network University of Wisconsin Breast Cancer Simulation Model. https://cisnet.flexkb.net/mp/pub/CISNET_ModelProfile_BREAST_UWISC_001_07232013_58567.pdf.

[25] Singletary S.E. (2003). Rating the risk factors for breast cancer. Ann. Surg..

[26] Chlebowski R.T., Rohan T.E., Manson J.E., Aragaki A.K., Kaunitz A.M., Stefanick M.L., Simon M.S., Johnson K.C., Wactawski-Wende J., O’sullivan M.J. (2015). Breast cancer after use of estrogen plus progestin and estrogen alone: Analyses of data from 2 women’s health initiative randomized clinical trials. JAMA Oncol..

[27] Ontario Ministry of Health and Long-Term Care Ontario Health Insurance Plan Schedule of Benefits and Fees. https://www.health.gov.on.ca/en/pro/programs/ohip/sob/.

[28] (2013). Ontario Breast Screening Program 2011 Report.

[29] Cheung M.C., Earle C.C., Rangrej J., Ho T.H., Liu N., Barbera L., Saskin R., Porter J., Seung S.J., Mittmann N. (2015). Impact of aggressive management and palliative care on cancer costs in the final month of life. Cancer.

[30] Guertin J.R., Feeny D., Tarride J.-E. (2018). Age- and sex-specific Canadian utility norms, based on the 2013–2014 Canadian Community Health Survey. CMAJ.

[31] Boswell-Purdy J., Flanagan W.M., Roberge H., Le Petit C., White K.J., Berthelot J.-M. (2007). Population health impact of cancer in Canada, 2001. Chronic Dis. Inj. Can..

[32] Alagoz O., Ergun M.A., Cevik M., Sprague B.L., Fryback D.G., Gangnon R.E., Hampton J.M., Stout N.K., Trentham-Dietz A. (2018). The University of Wisconsin Breast Cancer Epidemiology Simulation Model: An Update. Med. Decis. Mak..

[33] Mavaddat N., Barrowdale D., Andrulis I., Domchek S.M., Eccles D., Nevanlinna H., Ramus S.J., Spurdle A., Robson M., Sherman M. (2012). Pathology of breast and ovarian cancers among BRCA1 and BRCA2 mutation carriers: Results from the Consortium of Investigators of Modifiers of BRCA1/2 (CIMBA). Cancer Epidemiol. Biomark. Prev..

[34] Borgquist S., Anagnostaki L., Jirström K., Landberg G., Manjer J. (2007). Breast tumours following combined hormone replacement therapy express favourable prognostic factors. Int. J. Cancer.

[35] Flanagan W.M., McIntosh C.N., Le Petit C., Berthelot J.-M. (2006). Deriving utility scores for co-morbid conditions: A test of the multiplicative model for combining individual condition scores. Popul. Health Metr..

[36] Statistics Canada Deaths, by Cause: Malignant Neoplasm of Breast (C50), Canada, 2018 (Table: 13-10-0142-01). https://www150.statcan.gc.ca/t1/tbl1/en/cv.action?pid=1310014201.

[37] Moss S.M., Cuckle H., Evans A., Johns L., Waller M., Bobrow L. (2006). Effect of mammographic screening from age 40 years on breast cancer mortality at 10 years’ follow-up: A randomised controlled trial. Lancet.

[38] Moss S.M., Wale C., Smith R., Evans A., Cuckle H., Duffy S.W. (2015). Effect of mammographic screening from age 40 years on breast cancer mortality in the UK Age trial at 17 years’ follow-up: A randomised controlled trial. Lancet Oncol..

[39] Broek J.J.V.D., Van Ravesteyn N.T., Mandelblatt J.S., Huang H., Ergun M.A., Burnside E.S., Xu C., Li Y., Alagoz O., Lee S.J. (2018). Comparing CISNET Breast Cancer Incidence and Mortality Predictions to Observed Clinical Trial Results of Mammography Screening from Ages 40 to 49. Med. Decis. Mak..

[40] Moss S., Waller M., Anderson T.J., Cuckle H. (2005). Randomised controlled trial of mammographic screening in women from age 40: Predicted mortality based on surrogate outcome measures. Br. J. Cancer.

[41] Yong J.H., Mainprize J.G., Yaffe M.J., Ruan Y., Poirier A.E., Coldman A., Nadeau C., Iragorri N., Hilsden R.J., Brenner D.R. (2021). The impact of episodic screening interruption: COVID-19 and population-based cancer screening in Canada. J. Med. Screen.

[42] Kerlikowske K. (2010). Epidemiology of ductal carcinoma in situ. J. Natl. Cancer Inst..

[43] To T., Wall C., Baines C.J., Miller A.B. (2014). Is carcinoma in situ a precursor lesion of invasive breast cancer?. IJC.

[44] Nekhlyudov L., Habel L.A., Achacoso N., Jung I., Haque R., Collins L.C., Schnitt S.J., Quesenberry C.P., Fletcher S.W. (2012). Ten-year risk of diagnostic mammograms and invasive breast procedures after breast-conserving surgery for DCIS. J. Natl. Cancer Inst..

[45] Tuttle T.M., Jarosek S., Habermann E.B., Arrington A., Abraham A., Morris T.J., Virnig B.A. (2009). Increasing rates of contralateral prophylactic mastectomy among patients with ductal carcinoma in situ. J. Clin. Oncol..

[46] Coldman A.J., Phillips N. (2012). False-positive Screening Mammograms and Biopsies Among Women Participating in a Canadian Provincial Breast Screening Program. Can. J. Public Health.

[47] Carney P.A., Miglioretti D.L., Yankaskas B.C., Kerlikowske K., Rosenberg R., Rutter C.M., Geller B.M., Abraham L.A., Taplin S.H., Dignan M. (2003). Individual and Combined Effects of Age, Breast Density, and Hormone Replacement Therapy Use on the Accuracy of Screening Mammography. Ann. Intern. Med..

