# Supplementary material for: The OncoSim-Breast Cancer Microsimulation Model
Source: Curr Oncol. 2022 Mar 3;29(3):1619–33. doi: 10.3390/curroncol29030136 (PMC8947518; doi:10.3390/curroncol29030136)
Supplement: Supplementary file 1 [file curroncol-29-00136-s001.zip › Supplementary File S1.pdf]

Supplemental Methods, Tables and Figures for

The OncoSim-Breast Cancer Microsimulation model

Jean H. E. Yong, Claude Nadeau, Willian M. Flanagan, Andrew J. Coldman, Keiko Asakawa, Rochelle Garner, Natalie Fitzgerald, Martin Yaffe, and Anthony B. Miller

Table of content:

Section 1: Demography

Section 2: Natural history

Section 3: Cancer detection, staging and tumour biology

Section 4: Disease progression

Section 5: Screening

Section 6: Breast cancer healthcare costs

Section 7: Quality-adjusted life-years

Section 1: Demography

OncoSim simulates the Canadian population in 1872-2051 according to observed and projected demographics. Births (observed until 2019, projected thereafter) are simulated by birth year, sex, and province. Immigrants (observed until 2019, projected thereafter) are simulated by sex, age at time of immigration, and province of entry. The hazard of emigration and the return of emigrants to Canada are also simulated, stratified by sex, age, and province of departure or re-entry, as required. OncoSim also includes inter-provincial migration (observed until 2019, projected thereafter) which is simulated by sex, age, and outbound and in-bound province/territory. All-cause mortality (observed until 2004, projected thereafter) is affected by smoking (the model simulates smoking history for each individual).

Risk factors

We included key risk factors for oncogenesis and tumour growth: BRCA1/2 gene mutation, family history of breast cancer, and exposure to hormone replacement therapy (HRT).

Family history of breast cancer and BRCA1/2 gene mutation: Women are assigned to one of four categories (Table S1).

Table S1. Family history and BRCA1/2 gene mutation distribution

BRCA1/2 gene mutation	Family history	% of women[a]
BRCA1 and/or 2	All	0.1
No mutation	First-degree family history of breast cancer	10.0
No mutation	Second-degree family history of breast cancer	15.0
No mutation	No family history	74.9

[a] Proportion of women with BRCA1/2 gene mutation came from the Anglian Breast Cancer Study group, 2000; family history distribution was estimated from the Canadian National Breast Screening Study (CNBSS).[1] In that screening trial, ~27% of women aged 40-49 recruited to the trial had second-degree relative who had breast cancer.[1] We think

that the 27% prevalence is likely an overestimate because women with family history of breast cancer may have been more likely to self-refer to the screening trial.

Hormone replacement therapy use: Combination HRT use was modeled using data from the longitudinal National Population Health Survey (1994-2010). The use of combination HRT started in 1990, and varied by age, time period and geography. In OncoSim, current and former use of combination HRT affects the risk of developing breast tumour and the sensitivity of mammography screening.

Section 2: Natural history

The natural history of breast cancer is a process of occult tumour onset (oncogenesis), growth and spread. Tumours may be detected clinically as a result of physical symptoms, or asymptomatically as a result of screening. Tumour detection provides insight into the natural history process by identifying characteristics of tumours such as size, nodal status, and metastatic status according to age at detection.

OncoSim-Breast simulates ductal carcinoma *in situ* (DCIS) and invasive cancer: it does not simulate other types of *in situ* tumours (e.g. lobular carcinoma *in situ)* or other precursor tumours. Over the course of a simulated woman's lifetime, she may develop:

- a single DCIS tumour;
- a single invasive tumour;
- a single DCIS tumour that becomes invasive; or
- no breast tumour at all.

Ductal carcinoma *in situ* (DCIS) is treated as a marker of risk of invasive breast cancer, thus the presence of DCIS will modify the risk of developing invasive breast cancer, this allows for invasive cancer to occur in the absence (or presence) of DCIS.

Occult tumour onset

This equation describes the tumour onset (oncogenesis) in Oncosim:

$$\text{Oncogenesis rate} = \text{Baseline(age, year)} * RR_{PREDISPOSITION} * RR_{HRT} + AR_{PREVIOUS}$$

The *Baseline* term represents the assumed hazard of developing an occult tumour by age and year for all women in the population (Figure S1). Baseline rates are adjusted according to a woman's predisposition category ($RR_{PREDISPOSITION}$), her use of HRT (RR_{HRT}) and if she has previously had a tumour ($AR_{PREVIOUS}$). Since we only simulate one of each tumour type in this version of the natural history model, this last term is only applied for women who have previously had a DCIS tumour as an increased risk of developing an invasive cancer.

The most common way for an invasive cancer to be "born" is from a prior DCIS tumour. However, an invasive tumour can be born without an explicit in situ phase – these tumours may be seen/construed as having an in-situ phase that became invasive prior to reaching the 2mm threshold of the simulation and therefore escape expression in the model. Additionally, because OncoSim-Breast does not explicitly model in situ breast cancer other than DCIS, in cases where natural history models invasive breast cancer without a prior DCIS, these could conceptually arise out of other in situ tumours that are not DCIS. In OncoSim, the incidence varies by age group and time period. It was calibrated from the inputs in the Wisconsin Breast model to match the incidence data in the National Cancer Incidence Reporting System (1969-1991) and the Canadian Cancer Registry (1992-2013) (Figure S1). After a tumour is simulated, the model assigns the tumour type (DCIS vs. invasive) by age (Table S2).

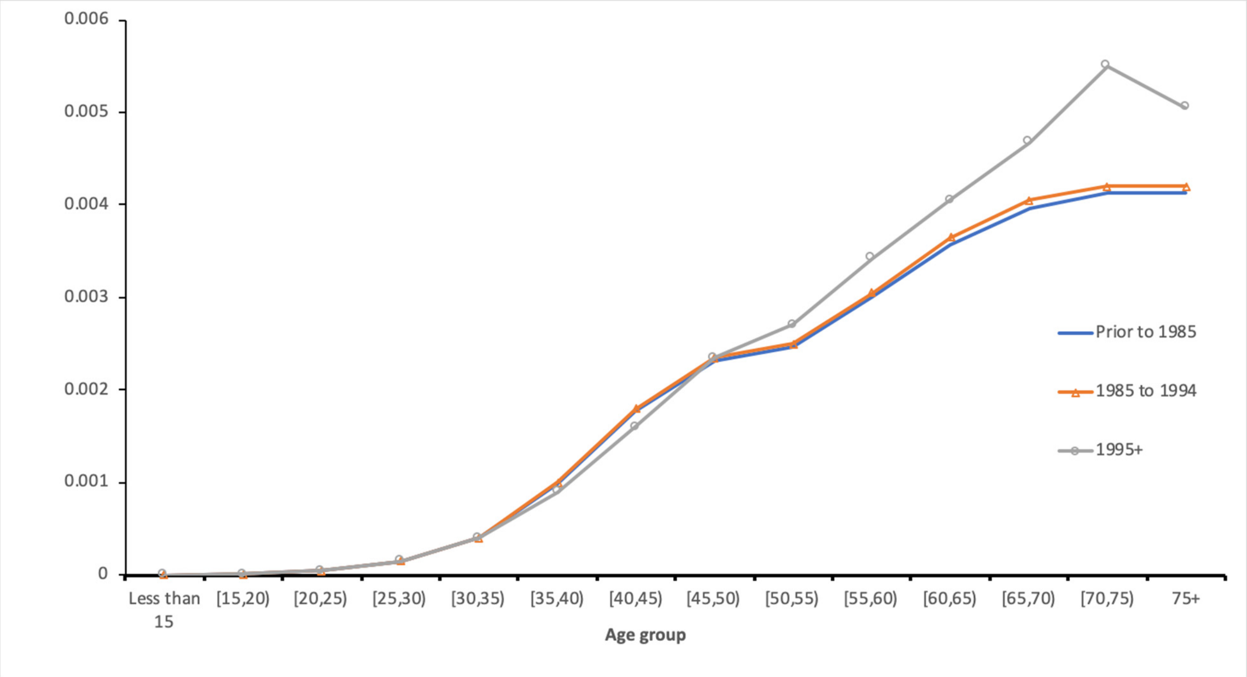

Figure S1. Incidence of 2mm occult tumours by age and time period (baseline)

Table S2. Distribution of tumour type by age

Age	0-54	55-64	65-69	70-79	80+
**DCIS**	19%	10%	16%	11%	2%
**invasive**	81%	90%	84%	89%	98%

Source: Calibration

Impact of family history and BRCA1/2 gene mutation on incidence: Women with BRCA1 and/or 2 gene mutation or family history of breast cancer have higher risk of breast cancer than those without the gene mutation nor family history (Table S3).

Table S3. Impact of family history and BRCA1/2 gene mutation on breast cancer incidence

BRCA1/2 gene mutation	Family history	Relative risk[a] Mean (variance)
BRCA1 and/or BRCA2 gene mutation	All	9.67 (0)
No mutation	First-degree family history of breast cancer	1.88 (4.2)
No mutation	Second-degree family history of breast cancer	1.41 (4.2)
No mutation	No family history	0.94 (4.2)

[a] Relative risk of developing occult tumour, as compared to average risk women, was modelled with a gamma distribution; the parameters (1.88 and 1.41) were calibrated from published estimates.[2]

Hormone replacement therapy use: In OncoSim, current and former use of combination HRT increases the risk of developing breast tumour (Table S4).

Table S4. Relative risk of developing breast cancer among HRT users, as compared to non-users.

Current users by duration use (years):	Relative risk[a]
**0 to 2**	1.3
**2 to 4**	1.8

**4 or more**	2.2
**Former users by time since quitting (years)**	
**0 to 20**	1.5
**20 or more**	1.0

[a] Relative risk of developing breast cancer was calibrated to match results of a study reporting impact of HRT on breast cancer risk.[3]

Table S5. Breast tumour growth equation coefficients

Predisposition category	Tumour type	Tumour class (% split within tumour type)	Growth Rate, α		Maximum diameter (cm), d_{max}		Propensity	
			Mean	Variance	Mean	Variance	Positive Node,⚜ ⚜$_N$	Meta-static, ⚜⚜$_M$
BRCA1/2	DCIS	A	1.36	1.73	7.75	0.00	0	0
	Invasive	B	1.36	1.73	7.75	0.00	1	1
Non-BRCA1/2	DCIS	C (96%)	0.43	3.48	2.54	2.62	0	0
		D (4%)	1.60	2.30	3.78	4.54	0	0
	Invasive	E (92%)	1.36	1.73	2.91	1.50	(1.2, 2.2)	(0.2, 0.2)
		F (8%)	2.23	0.71	9.01	0.90	(3.0, 0.1)	(0.02, 0.02)

Notes: ***a****. The notation (A,B) shown under ⚜$_N$ and ⚜$_M$ is used for Mean=A and Variance=B.* ***b****. BRCA1/2 is shown as two rows because of different propensity of DCIS and invasive cancer to generate nodes and metastasize, but in fact only one tumour growth equation was estimated.* ***c****. Non-BRCA1/2 tumour growth equations apply to women with and without family history so long as they do not have BRCA1/2.* ***d.*** *Non-BRCA1/2 DCIS tumours come in two classes (C and D) as do the invasive tumours (E and F). The split proportions shown were determined through calibration.*

Tumour growth

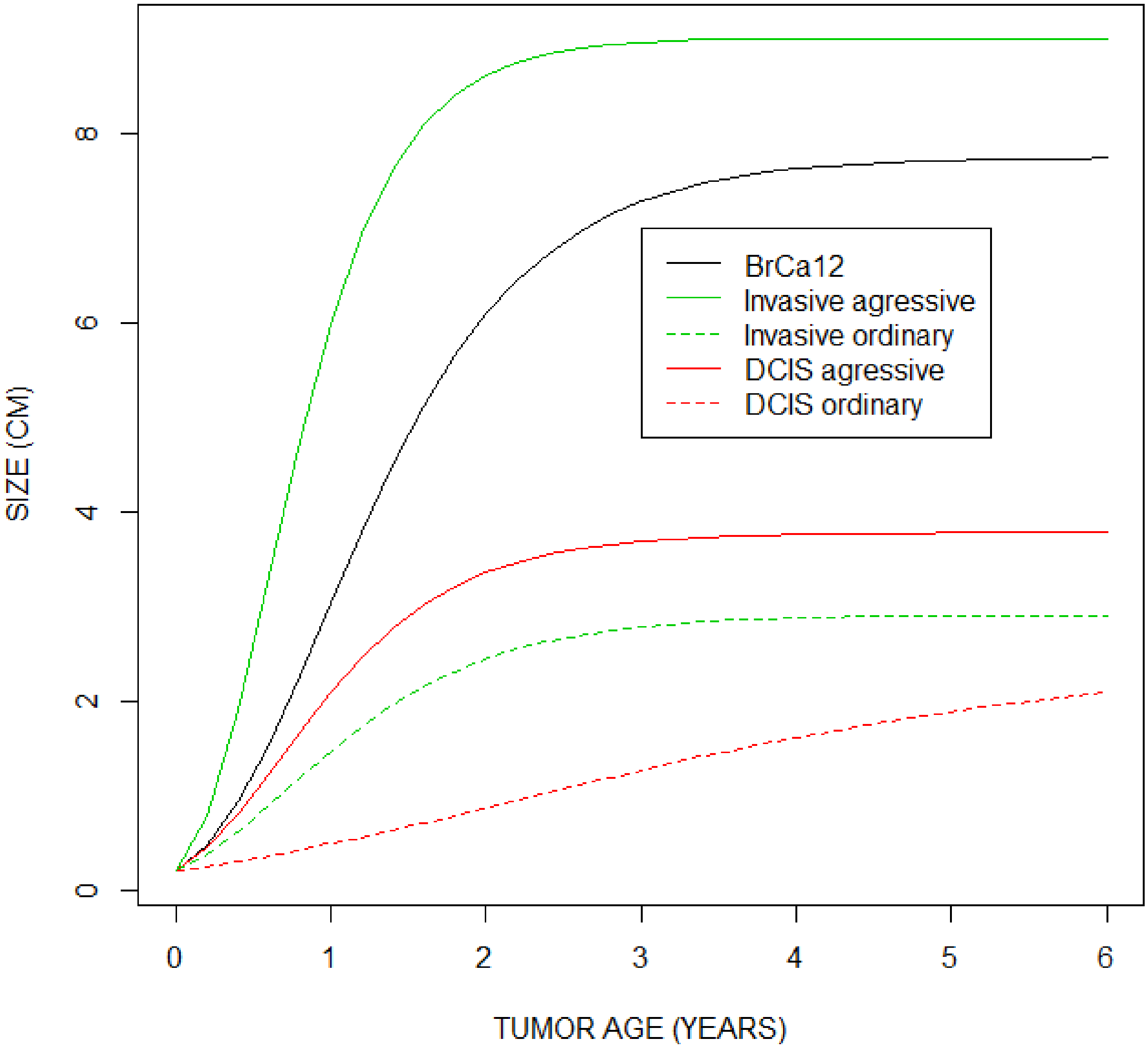

Figure S2. Tumour growth curves by tumour type, class (mean growth rate and mean maximum size)

Table S6. Tumour spread equation coefficients b1, b2 and b3

	**B1, i.e. time homogeneous term**	**B2, i.e. term multiplying the volume**	**B3, i.e. term multiplying the growth of the volume (derivative)**
**DCIS**	0.0696	0.0636	0.0002
**Breast cancer precursor other than DCIS**	0.0696	0.0636	0.0002
**Invasive breast cancer**	0.088325	0.099377	0.000223

Table S7. Annual hazard rate of metastasis as a function of breast tumor size and number of affected nodes, estimated through calibration

	**Annual hazard rate of metastasis**	
**Breast tumor size, cm**	**0-4 positive nodes**	**5 or more positive nodes**
**<=1.5**	0.0924	0.8910
**>1.5**	0.1068	0.8910

Section 3: Cancer detection, staging, tumour biology and disease progression

Table S8. Annual probability of clinical detection by tumour size

**Tumour size (cm)**	**0.2**	**0.3**	**0.9**	**1.4**	**1.9**	**2.8**	**3.7**	**4.7**	**7.5**	**8.4**
**Probability of tumour getting detected clinically, % per year**	0.7	0.7	7	8	30	55	75	80	100	100

Table S9. Stage definition for cancers without metastasis, based on American Joint Committee on Cancer (AJCC) 's classification*

**Node involvement**	**N0**	**N1mi**	**N1 (not N1mi)**	**N2**	**N3**
**Size Tis**	stage 0	stage 0	stage 0	stage 0	stage 0
**Size T1**	stage IA	stage IB	stage IIA	stage IIIA	stage IIIC
**Size T2**	stage IIA	stage IIB	stage IIB	stage IIIA	stage IIIC
**Size T3**	stage IIB	stage IIIA	stage IIIA	stage IIIA	stage IIIC
**Size T4**	stage IIIB	stage IIIB	stage IIIB	stage IIIB	stage IIIC

* Cancers with metastasis are classified as Stage IV

Table S10. Probability of a T4 tumour if there is no metastasis (cancers with metastasis are considered stage IV and do not need T and N information for staging)

Breast tumour size	Number of positive nodes	Probability of a T4 tumour
(-∞,1.0)	(-∞,1)	0.001275
	[1,2)	0.001762
	[2,3)	0.001687
	[3,4)	0.002073
	[4,5)	0.001666
	[5,10)	0.003274
	[10,∞)	0.003829
[1.0,2.0)	(-∞,1)	0.003424
	[1,2)	0.004725
	[2,3)	0.004526
	[3,4)	0.005559
	[4,5)	0.00447
	[5,10)	0.008759
	[10,∞)	0.010235
[2.0,5.0)	(-∞,1)	0.017143
	[1,2)	0.023538
	[2,3)	0.02256
	[3,4)	0.027597
	[4,5)	0.022286
	[5,10)	0.042939
	[10,∞)	0.049884
[5.0,∞)	(-∞,1)	0.095815
	[1,2)	0.127747
	[2,3)	0.122984
	[3,4)	0.147069
	[4,5)	0.121641
	[5,10)	0.214197
	[10,∞)	0.241842

Table S11. Distribution of Node involvement

Number of positive nodes	Node distribution	Tis	T1	T2	T3	T4
(-∞,1)	N0	1	99.5%	97.9%	87.7%	67.6%
	N1mi	0	0.1%	0.2%	0.9%	1.8%
	N1 (not N1mi)	0	0.3%	1.5%	9.2%	26.4%
	N2	0	0.0%	0.3%	1.1%	0.7%
	N3	0	0.0%	0.1%	1.0%	3.4%
[1,2)	N0	1	2.3%	0.6%	0.1%	0.0%
	N1mi	0	33.9%	23.8%	15.8%	11.7%
	N1 (not N1mi)	0	63.5%	75.1%	83.6%	87.8%
	N2	0	0.2%	0.3%	0.2%	0.0%
	N3	0	0.1%	0.2%	0.3%	0.4%
[2,3)	N0	1	1.0%	0.2%	0.0%	0.0%
	N1mi	0	9.7%	6.0%	3.7%	2.6%
	N1 (not N1mi)	0	88.3%	92.3%	94.7%	95.8%
	N2	0	0.3%	0.4%	0.3%	0.1%
	N3	0	0.7%	1.0%	1.3%	1.6%
[3,4)	N0	1	1.0%	0.2%	0.0%	0.0%
	N1mi	0	3.4%	2.1%	1.2%	0.9%
	N1 (not N1mi)	0	94.9%	96.8%	97.8%	98.1%
	N2	0	0.2%	0.2%	0.2%	0.0%
	N3	0	0.5%	0.6%	0.8%	1.0%
[4,5)	N0	1	0.0%	0.0%	0.0%	0.0%
	N1mi	0	1.0%	0.4%	0.4%	1.2%
	N1 (not N1mi)	0	2.7%	1.9%	3.0%	11.8%
	N2	0	95.3%	96.7%	94.6%	78.1%
	N3	0	1.0%	1.0%	1.9%	8.9%
[5,10)	N0	1	0.0%	0.0%	0.0%	0.0%
	N1mi	0	0.3%	0.1%	0.1%	0.3%
	N1 (not N1mi)	0	2.8%	2.0%	3.1%	12.1%
	N2	0	95.8%	96.8%	94.5%	77.1%
	N3	0	1.1%	1.1%	2.3%	10.5%
[10,∞)	N0	1	4.8%	0.8%	0.1%	0.0%
	N1mi	0	0.9%	0.4%	0.2%	0.1%

	N1 (not N1mi)	0	2.5%	1.9%	1.5%	1.3%
	N2	0	0.2%	0.3%	0.1%	0.0%
	N3	0	91.5%	96.6%	98.0%	98.6%

Section 4. Disease progression

Table S12. Transition probability from diagnosis to local recurrence - Weibull regression coefficients

**Stage**	**Parameter**	**Coefficients**
**Breast cancer, stage 0**	Intercept	4.6431
	Scale parameter	0.8418
**Breast cancer, node negative, small (<=1cm)**	Intercept	4.43
	Hormone positive (ER+ and/or PR+)	0.997
	Scale parameter	0.8467
**Breast cancer, node negative, larger (>1cm)**	Intercept	5.5438
	Year of diagnosis (continuous, centered)	-0.3354
	Grade low (I)	0.8437
	Grade moderate (II)	0.3555
	Non-screen detected	-0.7663
	Scale parameter	0.9414
**Breast cancer, node positive, low nodal burden**	Intercept	5.38
	Grade low (I)	1.9995
	Grade moderate (II)	1.2854
	Non-screen detected	-0.7506
	Scale parameter	1.0851
**Breast cancer, stage III**	Intercept	3.3078
	Age under 50 years (30-49)	0.8489
	Age between 50 and 69 years	1.2145
	Hormone positive (ER+ and/or PR+)	1.5373
	Scale parameter	1.3915

Table S13. Transition probability from diagnosis to distant recurrence – Weibull regression coefficients

Stage	Parameter	Coefficients
**Breast cancer, stage 0**	Intercept	6.2014
	Scale parameter	0.8367
**Breast cancer, node negative, small (<=1cm)**	Intercept	5.0368
	Age under 50 years (30-49)	1.6938
	Age between 50 and 69 years	1.5177
	Hormone positive (ER+ and/or PR+)	1.6537
	Non-screen detected	-1.3933
	Scale parameter	1.1749
**Breast cancer, node negative, larger (>1cm)**	Intercept	5.0876
	Year of diagnosis (continuous, centered)	-0.1384
	Grade low (I)	1.3492
	Grade moderate (II)	0.6077
	Interval cancer	-0.7501
	Never screened	-1.2192
	Scale parameter	1.0344
**Breast cancer, node positive, low nodal burden**	Intercept	2.5113
	Age under 50 years (30-49)	0.9812
	Age between 50 and 69 years	0.8384
	Grade low (I)	3.076
	Grade moderate (II)	1.8355
	Hormone positive (ER+ and/or PR+)	0.4184
	Non-screen detected	-0.0435
	Interaction: (Grade low)*(Age under 50)	0.4263
	Interaction: (Grade low)*(Age 50-69)	-0.3323
	Interaction: (Grade moderate)*(Age under 50)	-1.1584
	Interaction: (Grade moderate)*(Age 50-69)	-0.8897
	Interaction: (Grade low)*(Non-screen detected)	-1.8743
	Non-screen detected	-0.6759
	Scale parameter	0.9219
**Breast cancer, stage III**	Intercept	2.8814
	Grade low (I)	0.8306
	Grade moderate (II)	0.5848
	Hormone positive (ER+ and/or PR+)	0.3969
	Scale parameter	1.0504

Table S14. Transition probability from diagnosis to death – Weibull regression coefficients

Stage	Parameter	Coefficients
**Breast cancer, stage III**	Intercept	3.6024
	Year of diagnosis (continuous, centered)	-0.2895
	Age under 50 years (30-49)	1.7677
	Age between 50 and 69 years	1.2176
	Scale parameter	0.8975
**Breast cancer, stage IV**	Intercept	-0.3242
	Age under 50 years (30-49)	0.9169
	Age between 50 and 69 years	0.4807
	Grade low (I)	0.5579
	Grade moderate (II)	0.277
	Hormone positive (ER+ and/or PR+)	1.2041
	Her2 neu positive	1.2116
	Interaction: (Hormone positive) *(Her2 neu positive)	-1.1255
	Scale parameter	0.911

Table S15. Transition probability from local/regional recurrence to distant recurrence and death – Weibull regression coefficients

Cancer progression	Parameter	Coefficients
**Local recurrence to distant recurrence**	Intercept	-0.1778
	Stage at initial diagnosis (DCIS)	4.1839
	Stage at initial diagnosis (Node negative, small)	2.1066
	Stage at initial diagnosis (Node negative, large)	2.408
	Stage at initial diagnosis (Node positive)	1.0607
	Grade low (I)	0.9982
	Grade moderate (II)	1.5816
	Hormone positive (ER+ and/or PR+)	0.9887
	Scale parameter	1.8699
**Local recurrence to death**	Intercept	1.9457
	Year of diagnosis (continuous, centered)	-0.3915
	Age under 50 years (30-49)	2.7659
	Age between 50 and 69 years	0.8665
	Hormone positive (ER+ and/or PR+)	0.778
	Interval cancer	0.6067
	Never screened	-0.6766
	Scale parameter	0.8538

Table S16. Transition probability from distant recurrence to death – Weibull regression coefficients

Parameter	Coefficients
Intercept	-0.5918
Age under 50 years (30-49)	0.993
Age between 50 and 69 years	0.5249

Grade low (I)	0.295
Grade moderate (II)	0.433
Hormone positive (ER+ and/or PR+)	0.5567
Scale parameter	1.0774

Table S17. Survival curve adjustment for province/territory (relative risks)

	Stage 0	Node negative, small (<=1cm)	Node negative, larger (>1cm)	Node positive, low nodal burden	Stage III	Stage IV	Local/ regional recurrence	Distant recurrence
**Newfoundland and Labrador**	1	1.365	1.365	1.365	1.365	1.911	1.365	1.365
**Prince Edward Island**	1	1.239	1.239	1.239	1.239	1.735	1.239	1.239
**Nova Scotia**	1	1.208	1.208	1.208	1.208	1.691	1.208	1.208
**New Brunswick**	1	1.109	1.109	1.109	1.109	1.553	1.109	1.109
**Quebec**	1	1.296	1.296	1.296	1.296	1.814	1.296	1.296
**Ontario**	1	1.065	1.065	1.065	1.065	1.491	1.065	1.065
**Manitoba**	1	1.296	1.296	1.296	1.296	1.814	1.296	1.296
**Saskatchewan**	1	1.201	1.201	1.201	1.201	1.681	1.201	1.201
**Alberta**	1	1.032	1.032	1.032	1.032	1.445	1.032	1.032
**British Columbia**	1	1	1	1	1	1.4	1	1
**Yukon**	1	1.365	1.365	1.365	1.365	1.911	1.365	1.365
**North West Territories and Nunavut**	1	1.365	1.365	1.365	1.365	1.911	1.365	1.365

Section 5: Screening

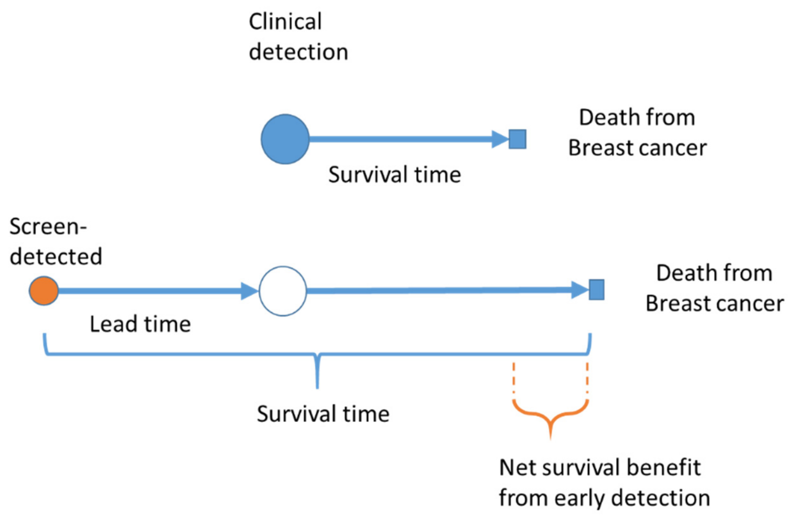

Figure S3. Diagram to show how screening affects survival (for illustrative purpose only)

Screening modality

The model allows screening modality to vary by province and time period. In the base case scenario, we assumed that all provinces use digital radiography mammography in 2018 and beyond. The model also includes emerging screening modalities, such as tomosynthesis and ultrasound, to accommodate changes in screening modality in the future. To evaluate a new screening modality, users can change the distribution of screening modality and test performance. The historical screening test distribution for each province came from the Canadian Breast Cancer Screening Database (Figure S4).

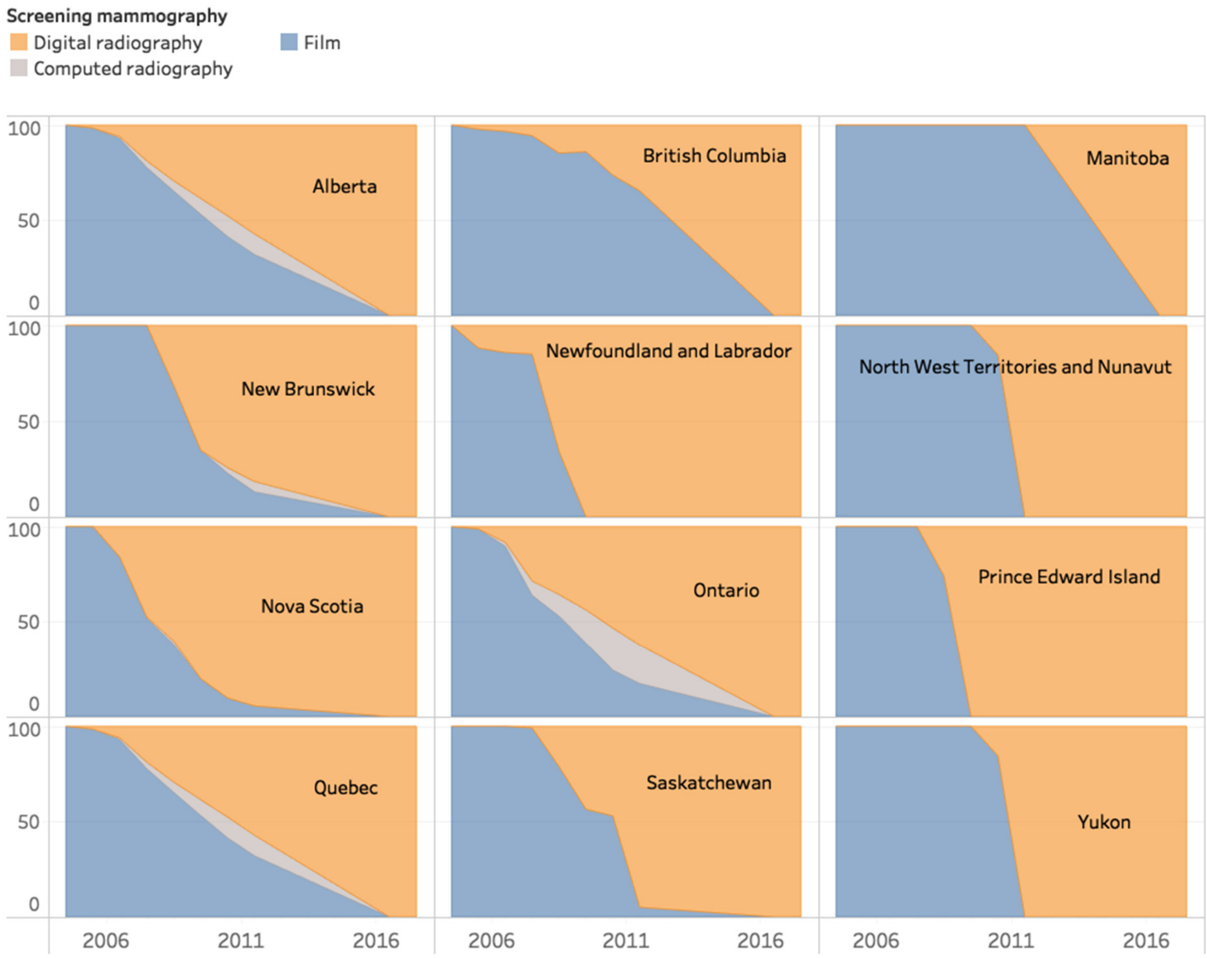

Figure S4. Historical mammography distribution by province and year

Sensitivity

Sensitivity of screening test varies by time period, type of mammography, screen sequence, age group and tumour size (Figure S5). The sensitivity estimates were calibrated from the inputs in the Wisconsin Breast model to match the incidence data in the National Cancer Incidence Reporting System (1969-1991) and the Canadian Cancer Registry (1992-2013), positive predictive value, sensitivity and tumour detection rate in the Canadian Breast Cancer Screening Database (CBCSD) in 2007-2008.

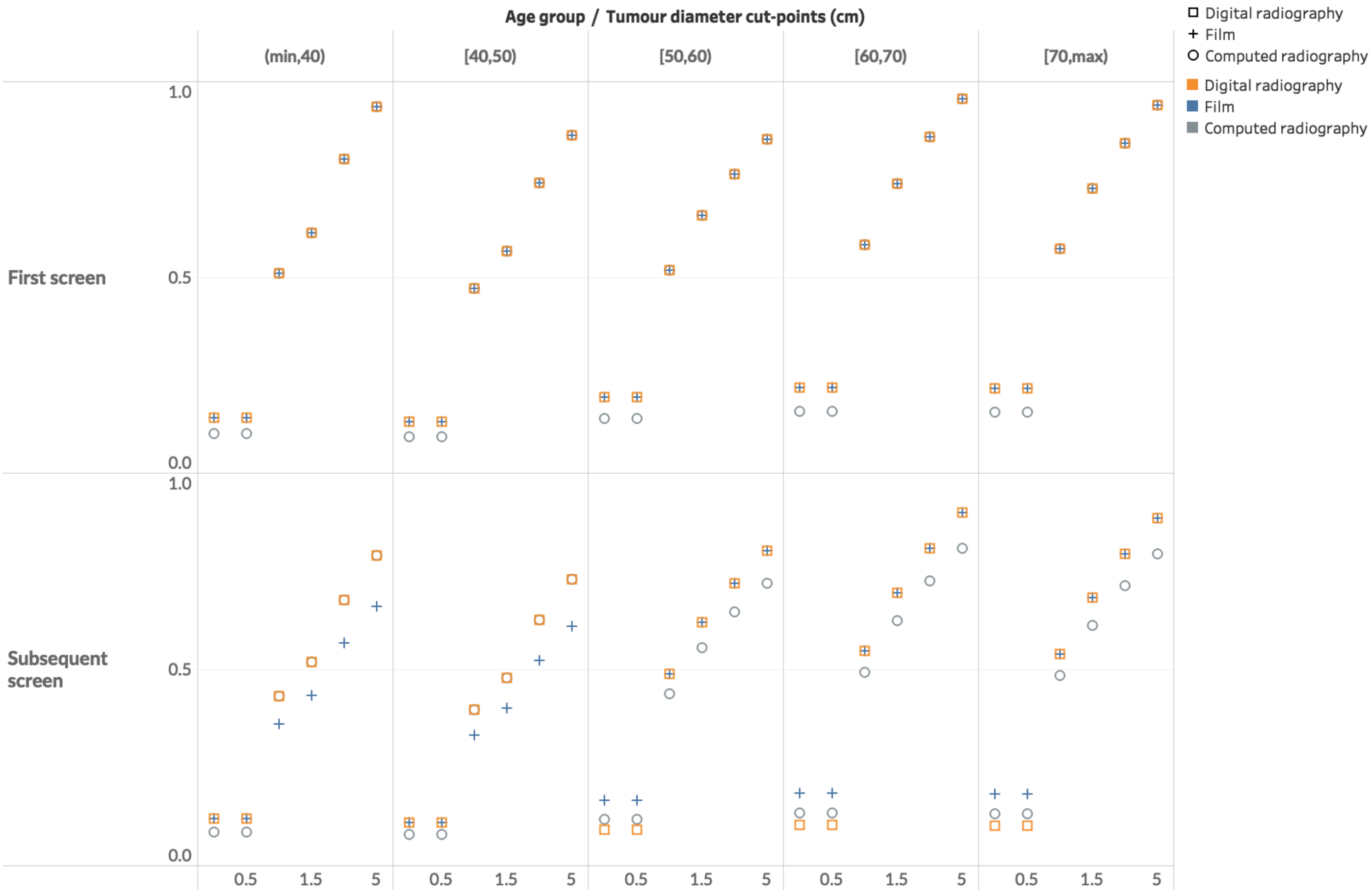

Figure S5. Sensitivity of mammography in 2010, by age group, tumour diameter cut-points, type of mammography, and screen sequence

Specificity

The model allows users to set specificity of screening test by screen sequence, time since previous screen, age group, time period, screening modality and breast density. In the base case scenario, specificity of screening test varies by screen sequence and age (Table S18), but not by modality and breast density.

Table S18. Specificity of mammography screening by screen sequence and age

Screen sequence	Time since previous screen	Age group				
		Under 40	40-49	50-59	60-69	70+
First screen	-	0.856	0.856	0.876	0.894	0.916
Subsequent screen	< 30 months	0.937	0.937	0.945	0.952	0.956
	> 30 months	0.912	0.912	0.919	0.926	0.930

Source: *Calibration started with estimates from Coldman et al. to match the 2008 abnormal call rate reported in the Canadian Breast Cancer Screening Database.*[11]

Impact of HRT use on Sensitivity and Specificity

Sensitivity is reduced by 15% for women aged 70 or older actively taking combination HRT. Specificity is reduced by 0.5% for women aged 50-59 actively taking combination HRT, by 2.5% for women aged 60-69 actively taking combination HRT, and by 3.5% in women aged 70 and older actively taking combination HRT. These estimates were based on Carney et al. (2003).[12]

Costs of screening and workup

The base case scenario assumed each screening mammogram costs $64.15, which includes the technical and professional components of physician fees for performing a bilateral mammogram for asymptomatic individuals.[13] We assumed all abnormal findings are followed-up with diagnostic imaging. The costs were estimated using abnormal workup distribution data from the Ontario Breast Screening Program 2011, Canadian Breast Cancer Screening Database 2004-2008 and the technical and professional components of physician fees for performing the workup.[13,14]

Table S19. Distribution and weighted cost of follow-up workup for women with abnormal mammogram results

Diagnostic procedures	Weighted cost[a]	False positive	True positive
**Imaging only[b]**	$68.32	91.1%	0
**Imaging + core biopsy and/or fine needle aspiration[c]**	$181.42	7.7%	89.3%
**Imaging + open biopsy[d]**	$431.88	1.2%	10.7%
**Weighted cost**		$81.56	$208.23

[a]OHIP physician fees[13]

[b]18.6% receive mammogram only, 22.1% receive ultrasound only, 58.4% receive mammogram and ultrasound, 0.9% receive mammogram, ultrasound and magnetic resonance imaging (MRI), estimated from Canadian Breast Cancer Screening Database 2004-2008.

[c]83.6% receive core biopsy only, 16.4% receive core biopsy and fine needle aspiration, estimated from Canadian Breast Cancer Screening Database 2004-2008.

[d]75.4% receive open biopsy with fine wire localization, estimated from Canadian Breast Cancer Screening Database 2004-2008.

Section 5: Disease progression

Figure S6 shows the average simulated survival time by stage and age in the base case scenario (OncoSim v3.3.3).

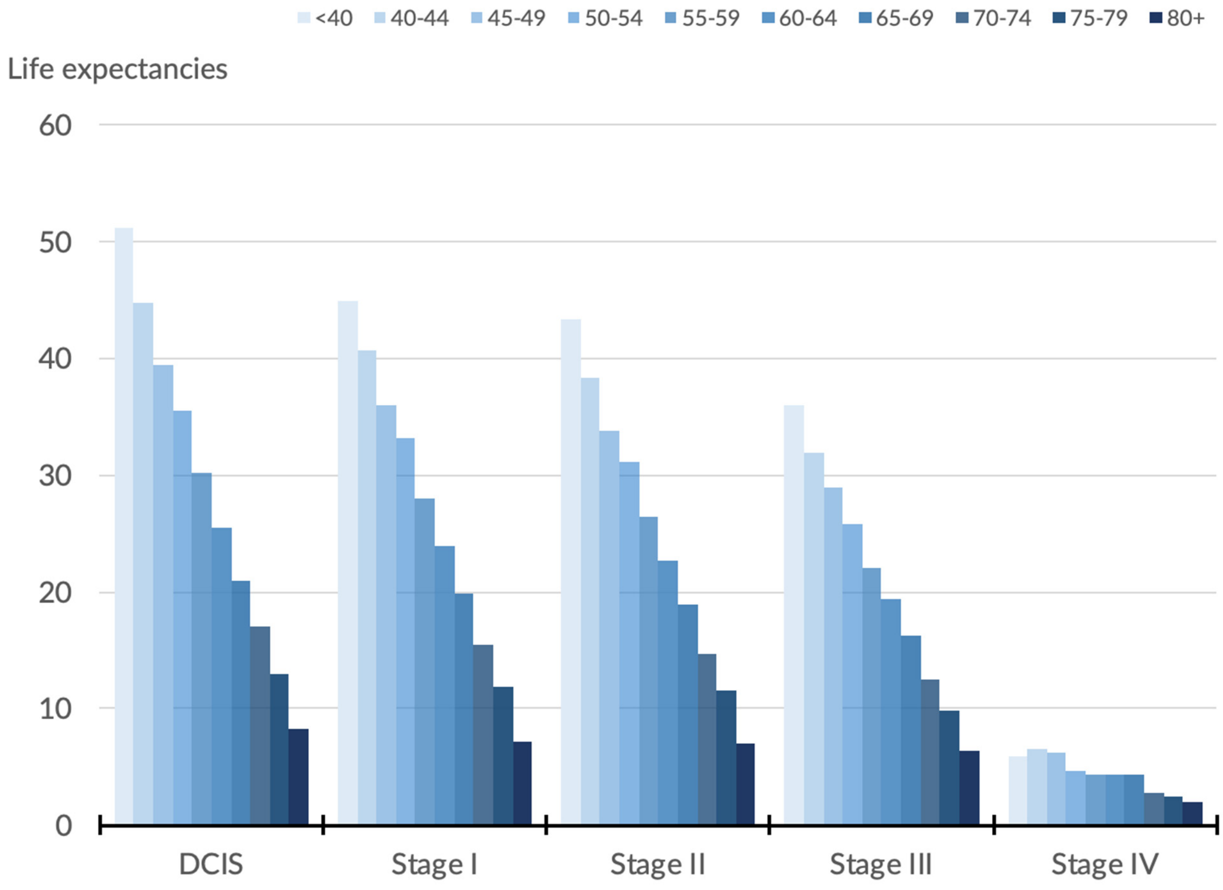

Figure S6. Projected average survival time (years) by age and stage at diagnosis

Section 6: Breast cancer healthcare costs

Healthcare costs associated with breast cancer were estimated from the perspective of the public healthcare payer, e.g. Ministry of Health. The model estimates lifetime costs of breast cancer by phase of care.

First 18 months: Costs for the first 18 months after diagnosis or cancer recurrence ("acute treatment costs") were specific to breast cancer treatments. The costs were estimated from a retrospective analysis of healthcare administrative data at the Institute for Clinical Evaluative Sciences (ICES): cancer registry (Cancer Care Ontario), hospitalizations (inpatient, day surgery), physician billings (OHIP), Ontario Drug Benefit program, New Drug Funding Program, and Activity Level Reporting data. The analyses included 11,164 women diagnosed with invasive breast cancer in 2010 or the first 6 months of 2011 where the following cancer characteristics were known: (i) tumour size, (ii) disease stage (collaborative staging), (iii) estrogen receptor and progesterone receptor status (negative, positive), (iv) HER2 status, and (v) tumour grade. The analysis included costs incurred during the first 18 months after diagnosis: breast cancer surgery, radiation treatment, chemotherapy, hormonal treatments, imaging tests, and oncology physician fees. Costs varied by stage and age at diagnosis, molecular subtypes, and grade. Upon recurrence, patients incur additional treatment costs. Since the treatment cost inputs include many types of costs and are broken down by many subgroups, it is not feasible to show all input tables in this document. Figure S7 shows an overview of the retrospective database analysis cohort and the aggregated treatment patterns (surgery, radiation therapy and chemotherapy) by stage and age.

Continuing care: After 18 months, patients incur continuing care costs, which included follow-up care with oncology physicians and primary care physicians, laboratory tests and imaging for surveillance.[15,16] Probabilities and frequency of follow-up care were estimated using clinical expert opinions; costs were then estimated using OHIP schedule of benefits[13]. The model allows continuing care costs to vary by age group, stage, molecular subtypes, grade, and time after diagnosis. Five years after diagnosis with stages 0-III cancer, we assumed that the continuing

care costs would decline to include minimal surveillance physician visits and imaging tests. We made this assumption to avoid over-estimating treatment costs; most patients diagnosed with 0-III breast cancer live for many years after diagnosis, and most costing studies did not have sufficient follow-up time to accurately estimate their long-term healthcare costs.

Terminal care: Those who die from breast cancer costs incur terminal care costs in the last three months. We estimated the end-of-life care costs by conducting additional administrative database analyses, building upon a previously published end-of-life care costing study[17]. The costs were estimated from a cohort of 1904 patients diagnosed with breast cancer in 2010-2011 and died from breast cancer before March 31, 2014. In addition to the type of costs included in the acute treatment costs, the costs for terminal care include acute hospitalizations, emergency department visits, home care, long-term care, complex continuing care, and others (mental health, dialysis, rehabilitation and devices).

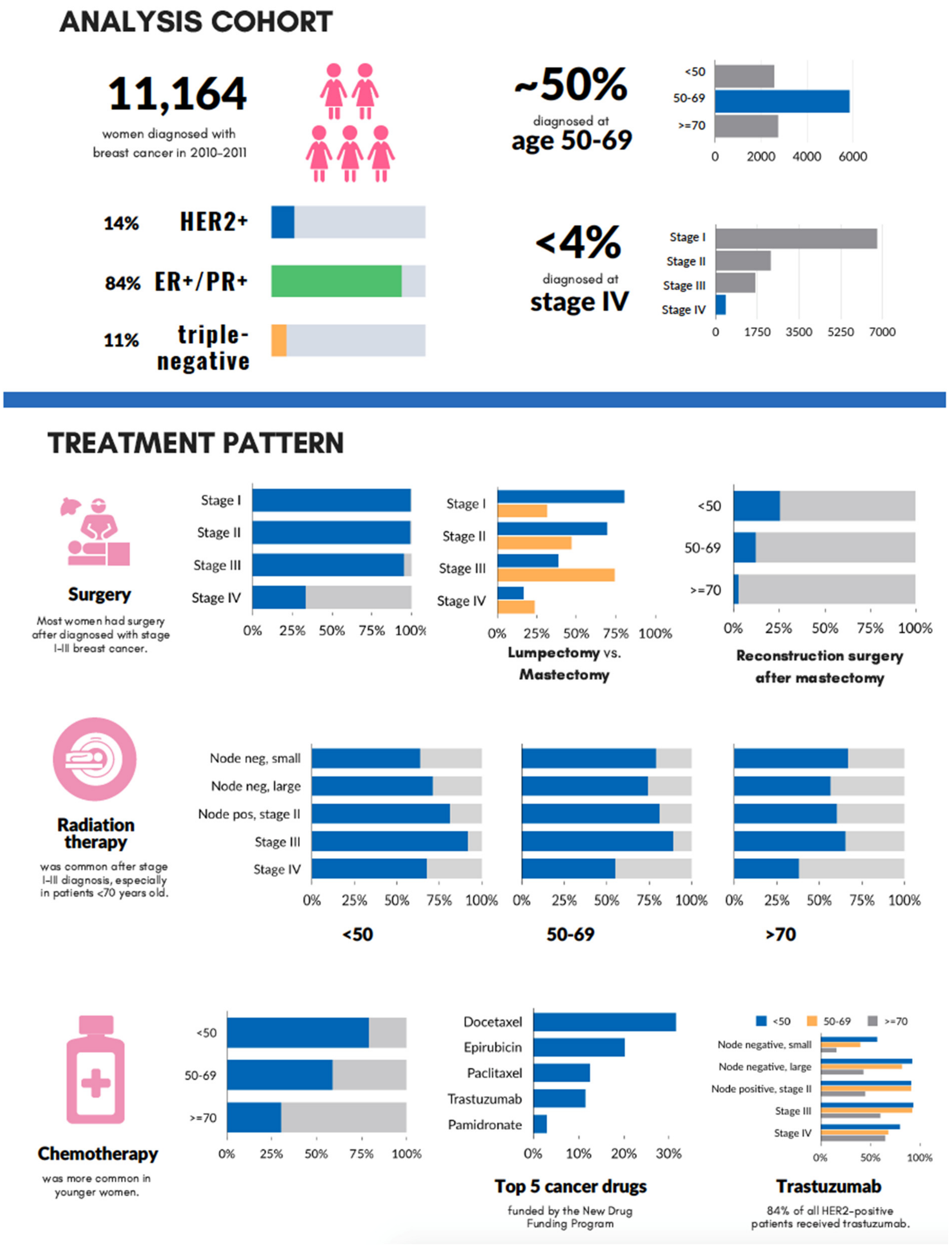

Figure S7. Infographic shows an overview of the retrospective database analysis cohort and the aggregated treatment patterns (surgery, radiation therapy and chemotherapy) by stage and age

Table S20. Projected lifetime costs associated with breast cancer by stage at diagnosis (2019 CAD$), average per case

Breast cancer stage at initial diagnosis	Lifetime costs, per case
DCIS	16,900
Stage IA	26,500
Stage IB	38,200
Stage IIA	34,800
Stage IIB	42,800
Stage III	54,100
Stage IV	82,100
All	36,000

Section 7: Quality-adjusted life-years

Canadian general population

To calculate quality-adjusted life-years for individuals who do not have a breast cancer diagnosis, the model multiplies each life-year with the age-sex specific utility scores of the Canadian general population. The utility scores were measured using Health Utilities Index Mark 3 and estimated from over 30 million community-dwelling Canadians (98% of the Canadian population) in the 2013-2014 Canadian Community Health Survey.[16]

Table S21. Age-sex specific utility weight of the Canadian general population

AGE_GROUP	Female	Male
**[min,5[**	1	1
**[5,10[**	0.95	0.95
**[10,15[**	0.93	0.93
**[15,20[**	0.879	0.892
**[20,25[**	0.89	0.892
**[25,30[**	0.902	0.902
**[30,35[**	0.893	0.899
**[35,40[**	0.89	0.898
**[40,45[**	0.874	0.901
**[45,50[**	0.862	0.873
**[50,55[**	0.842	0.856
**[55,60[**	0.83	0.85
**[60,65[**	0.841	0.842
**[65,70[**	0.837	0.848
**[70,75[**	0.831	0.841
**[75,80[**	0.778	0.809
**[80,85[**	0.736	0.748
**[85,90[**	0.616	0.682
**[90,95[**	0.616	0.682
**[95,max]**	0.616	0.682

Breast cancer

The breast cancer-specific utility scores came from a utility study that derived utility scores by classifying the impact of a health state across eleven attributes (each with four to five levels) using the CLAssification and MEasurement System of Functional Health (CLAMES).[17] To use the scores from that study, we made the following assumptions:

- We considered DCIS and breast cancer stages I, II, and III as cancers with very good prognosis, and breast cancer stage IV as metastatic cancer (utility score: 0.439).
- When individuals are receiving acute or continuing care, we assumed the utility scores are multiplicative.[18] For example, the health state utility of an individual diagnosed with stage IV cancer receiving chemotherapy = utility of metastatic cancer * utility of chemotherapy moderate toxicity

Table S22. Breast cancer-specific preference score

**Treatment phase**	**Preference score**
**Stage I-III breast cancer**	
**Diagnosis**	0.891
**Surgery and immediate follow-up**	0.652
**Radiotherapy**	0.696
**Chemotherapy**	0.661
**Anti-HER2 neu treatment**	0.668
**Hormonal therapy**	0.798
**No active treatment**	0.906
**Stage IV breast cancer**	
**Diagnosis**	0.439
**Surgery and immediate follow-up**	0.321
**Radiotherapy**	0.343
**Chemotherapy**	0.326
**Anti-HER2 neu treatment**	0.329
**No active treatment**	0.484

**Terminal care**	0.179

References

1. Miller AB, Baines CJ, To T, Wall C. Canadian National Breast Screening Study: 1. Breast cancer detection and death rates among women aged 40 to 49 years. CMAJ : Canadian Medical Association journal = journal de l'Association medicale canadienne 1992; 147(10): 1459-76.
2. Singletary SE. Rating the risk factors for breast cancer. Annals of surgery 2003; 237(4): 474.
3. Chlebowski RT, Rohan TE, Manson JE, et al. Breast cancer after use of estrogen plus progestin and estrogen alone: analyses of data from 2 women's health initiative randomized clinical trials. JAMA oncology 2015; 1(3): 296-305.
4. Alagoz O, Ergun MA, Cevik M, et al. The University of Wisconsin Breast Cancer Epidemiology Simulation Model: An Update. Med Decis Making 2018; 38(1_suppl): 99s-111s.
5. Kerlikowske K. Epidemiology of ductal carcinoma in situ. Journal of the National Cancer Institute Monographs 2010; 2010(41): 139-41.
6. To T, Wall C, Baines CJ, Miller AB. Is carcinoma in situ a precursor lesion of invasive breast cancer? International journal of cancer 2014; 135(7): 1646-52.
7. Nekhlyudov L, Habel LA, Achacoso N, et al. Ten-year risk of diagnostic mammograms and invasive breast procedures after breast-conserving surgery for DCIS. Journal of the National Cancer Institute 2012; 104(8): 614-21.
8. Tuttle TM, Jarosek S, Habermann EB, et al. Increasing rates of contralateral prophylactic mastectomy among patients with ductal carcinoma in situ. Journal of clinical Oncology 2009; 27(9): 1362-7.
9. Mavaddat N, Barrowdale D, Andrulis I, et al. HEBON; EMBRACE; GEMO Study Collaborators; kConFab Investigators; SWE-BRCA Collaborators; Consortium of Investigators of Modifiers of BRCA1/2. Pathology of breast and ovarian cancers among BRCA1 and BRCA2 mutation carriers: results from the Consortium of Investigators of Modifiers of BRCA1/2 (CIMBA). Cancer Epidemiol Biomarkers Prev 2012; 21(1): 134-47.

10. Borgquist S, Anagnostaki L, Jirström K, Landberg G, Manjer J. Breast tumours following combined hormone replacement therapy express favourable prognostic factors. International journal of cancer 2007; 120(10): 2202-7.
11. Coldman AJ, Phillips N. False-positive Screening Mammograms and Biopsies Among Women Participating in a Canadian Provincial Breast Screening Program. Canadian Journal of Public Health 2012; 103(6): e420-e4.
12. Carney PA, Miglioretti DL, Yankaskas BC, Kerlikowske K, Rosenberg R, Rutter CM, Geller BM, Abraham LA, Taplin SH, Dignan M, Cutter G, Ballard-Barbash R. 2003. Individual and Combined Effects of Age, Breast Density, and Hormone Replacement Therapy Use on the Accuracy of Screening Mammography. Annals of Internal Medicine, 138(3):168-175.

13. Ontario Ministry of Health and Long-Term Care. Ontario Health Insurance Plan Schedule of Benefits and Fees.
14. Cancer Care Ontario. Ontario Breast Screening Program 2011 Report. Toronto, Canada, 2013.
15. Cheung MC, Earle CC, Rangrej J, et al. Impact of aggressive management and palliative care on cancer costs in the final month of life. Cancer 2015; 121(18): 3307-15.
16. Guertin JR, Feeny D, Tarride J-E. Age-and sex-specific Canadian utility norms, based on the 2013–2014 Canadian Community Health Survey. *Cmaj.* 2018;190:E155-E61.
17. Boswell-Purdy J, Flanagan WM, Roberge H, Le Petit C, White KJ, Berthelot J-M. Population health impact of cancer in Canada, 2001. *Chronic Diseases and Injuries in Canada.* 2007;28.
18. Flanagan WM, McIntosh CN, Le Petit C, Berthelot J-M. Deriving utility scores for co-morbid conditions: a test of the multiplicative model for combining individual condition scores. Population Health Metrics 2006; 4:13.
